# Supplementary material for: A refined picture of the native amine dehydrogenase family revealed by extensive biodiversity screening
Source: Nat Commun. 2024 Jun 10;15:4933. doi: 10.1038/s41467-024-49009-2 (PMC11164908; doi:10.1038/s41467-024-49009-2)
Supplement: Supplementary file 1 — Supplementary Information [file 41467_2024_49009_MOESM1_ESM.pdf]

Supplementary Information

A refined picture of the native Amine Dehydrogenase family revealed by extensive biodiversity screening

Eddy Elisée,[1] Laurine Ducrot,[1] Raphaël Méheust,[1] Karine Bastard,[2] Aurélie Fossey-Jouenne,[1] Gideon Grogan,[3] Eric Pelletier,[1] Jean-Louis Petit,[1] Mark Stam,[1] Véronique de Berardinis,[1] Anne Zaparucha,[1] David Vallenet,[1]* Carine Vergne-Vaxelaire,[1]*

[1] Génomique Métabolique, Genoscope, Institut François Jacob, CEA, CNRS, Univ Evry, Université Paris-Saclay, 91057 Evry, France
[2] School of Pharmacy, Faculty of Medicine and Health, University of Sydney, Sydney N.S.W. 2006, Australia
[3] York Structural Biology Laboratory, Department of Chemistry, University of York, Heslington, York YO10 5DD, UK
*carine.vergne@genoscope.cns.fr, vallenet@genoscope.cns.fr

Supplementary Tables....2
Supplementary Figures 11
Supplementary Discussion 34
Supplementary References 35

Supplementary Tables

Supplementary Table 1. Metagenomic and genomic databases used in this study.

Resources	Details
UniProtKB SwissProt	https://ftp.uniprot.org/pub/databases/uniprot/previous_releases/release-2020_02/
	562,253 sequences
	266.2 MiB (release 2020_02)
UniProtKB TrEMBL	https://ftp.uniprot.org/pub/databases/uniprot/previous_releases/release-2020_03/
	184,998,855 sequences
	81.6 GiB (release 2020_03)
GEM (Genomes from Earth's Microbiomes)	https://portal.nersc.gov/GEM/protclusts/
	111,428,992 sequences
	25 GiB (download 2021_02)
OM-RGC (Ocean Microbial Reference Gene Catalog)	https://ocean-microbiome.embl.de/companion.html
	46,828,091 sequences
	15.4 GiB (download 2020_05)
MetDB (Marine Eukaryotes Transcriptomes)	https://metdb.sb-roscoff.fr/metdb/
	16,231,949 sequences
	7.4 GiB (download 2020_07)
SMAGs (Tara Oceans Eukaryote Metagenome Assembled Genomes)	https://www.genoscope.cns.fr/tara/
	10,207,435 sequences
	3.6 GiB (download 2020_09)
IGC (Integrated Gene Catalog of Human gut)	https://db.cngb.org/microbiome/genecatalog/genecatalog_human/
	9,878,647 sequences
	8.9 GiB (download 2020_07)
UHGP (Unified Human Gastrointestinal Protein)	http://ftp.ebi.ac.uk/pub/databases/metagenomics/mgnify_genomes/human-gut/v1.0/uhgp_catalogue/uhgp-100.tar.gz
	170,602,708 sequences
	67.3 GiB (release 2020_01)
MGnify (EMBL-EBI)	http://ftp.ebi.ac.uk/pub/databases/metagenomics/peptide_database/2019_05/
	1,106,951,200 sequences
	133.6 GiB (release 2019_05)
MATOUv2 (Tara Oceans Eukaryote Gene Catalog)	https://www.genoscope.cns.fr/tara/
	950,799,894 sequences
	174.1 GiB (download 2020_05)

Supplementary Table 2. Pfam annotations (version 35.0) of the ref-AmDH family (17,959 sequences). Main annotations for the C- and N-terminus are highlighted in bold.

Occurrence	Pfam	*Pfam description*
C-terminus		
**15536**	**PF19328**	***2,4-diaminopentanoate dehydrogenase C-terminal domain***
2	PF01381	*Helix-turn-helix*
2	PF00572	*Ribosomal protein L13*
2	PF00466	*Ribosomal protein L10*
1	PF18516	*RuvC nuclease domain*
1	PF18510	*Nuclease domain*
1	PF13385	*Concanavalin A-like lectin/glucanases superfamily*
1	PF10996	*Beta-Casp domain*
1	PF07501	*G5 domain*
1	PF06144	*DNA polymerase III, delta subunit*
1	PF02786	*Carbamoyl-phosphate synthase L chain, ATP binding domain*
1	PF02637	*GatB domain*
1	PF02562	*PhoH-like protein*
1	PF00815	*Histidinol dehydrogenase*
1	PF00682	*HMGL-like*
1	PF00589	*Phage integrase family*
1	PF00490	*Delta-aminolevulinic acid dehydratase*
1	PF00176	*SNF2-related domain*
1	PF00158	*Sigma-54 interaction domain*
1	PF00005	*ABC transporter*
N-terminus		
**8527**	**PF01113**	***Dihydrodipicolinate reductase, N-terminus***
167	PF02629	*CoA binding domain*
81	PF01408	*Oxidoreductase family, NAD-binding Rossmann fold*
32	PF01118	*Semialdehyde dehydrogenase, NAD binding domain*
27	PF03447	*Homoserine dehydrogenase, NAD binding domain*
25	PF03435	*Saccharopine dehydrogenase NADP binding domain*
8	PF02558	*Ketopantoate reductase PanE/ApbA*
7	PF03446	*NAD binding domain of 6-phosphogluconate dehydrogenase*
5	PF02254	*TrkA-N domain*
1	PF18952	*Family of unknown function (DUF5696)*
1	PF16745	*RsgA N-terminal domain*
1	PF16396	*Domain of unknown function (DUF5005)*
1	PF13411	*MerR HTH family regulatory protein*
1	PF10604	*Polyketide cyclase / dehydrase and lipid transport*
1	PF04230	*Polysaccharide pyruvyl transferase*
1	PF03952	*Enolase, N-terminal domain*
1	PF03807	*NADP oxidoreductase coenzyme F420-dependent*
1	PF03193	*RsgA GTPase*
1	PF02880	*Phosphoglucomutase/phosphomannomutase, alpha/beta/alpha domain III*
1	PF02784	*Pyridoxal-dependent decarboxylase, pyridoxal binding domain*
1	PF02775	*Thiamine pyrophosphate enzyme, C-terminal TPP binding domain*
1	PF01915	*Glycosyl hydrolase family 3 C-terminal domain*
1	PF01839	*FG-GAP repeat*
1	PF01583	*Adenylylsulphate kinase*
1	PF01580	*FtsK/SpoIIIE family*
1	PF01488	*Shikimate / quinate 5-dehydrogenase*
1	PF01451	*Low molecular weight phosphotyrosine protein phosphatase*
1	PF00724	*NADH:flavin oxidoreductase / NADH oxidase family*
1	PF00535	*Glycosyl transferase family 2*
1	PF00440	*Bacterial regulatory proteins, tetR family*
1	PF00294	*pfkB family carbohydrate kinase*
1	PF00226	*DnaJ domain*
1	PF00133	*tRNA synthetases class I (I, L, M and V)*
1	PF00072	*Response regulator receiver domain*
1	PF00036	*EF hand 146*

Supplementary Table 3. Number of AmDH models successfully clustered using the ASMC method.

ASMC groups	Former ASMC [Mayol *et al.*]	New ASMC [this publication]
G1	271	1,427
G2	256	1,970
G3	32	56
G4	265	5,652
G5	1,187	658
Total	*2,011*	*9,763*

Supplementary Table 4. Docking results for hypothesized G1 substrates into the protein E1QRK4 (UniProt ID).

Docked compounds	Energy of binding* (kcal mol^{-1}) and key interactions	Tested compounds
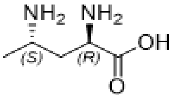 **1b** (2*R*,4*S*)-2,4-diaminopentanoic acid	**-5.07** γ-NH_2 oriented towards E103 and COOH oriented towards a positively charged area (R162-R169)	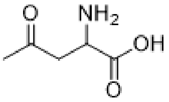 **1a** 2-amino-4-oxopentanoic acid
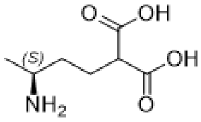 (*S*)-2-(3-aminobutyl)malonic acid	**-3.79** NH_2 oriented towards E103 and C2-COOH oriented towards a positively charged area (R162-R169)	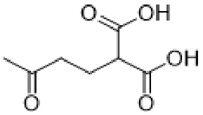 2-(3-oxobutyl)malonic acid
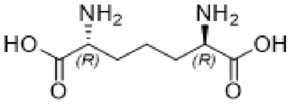 (2*R*,6*R*)-2,6-diaminoheptanedioic acid (= diaminopimelate)	**-3.37** NH_2 oriented towards E103 and COOH close to R169	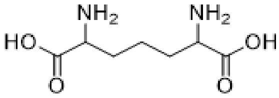 racemic diaminopimelate
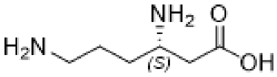 (*S*)-3,6-diaminohexanoic acid (= L-beta-lysine)	**-4.98** ω-NH_2 oriented towards E103 and COOH oriented towards a positively charged area (R162-R169)	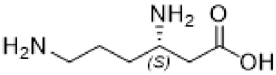 L-beta-lysine
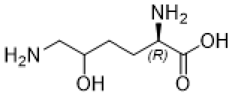 (2*R*)-2,6-diamino-5-hydroxyhexanoic acid	**-5.19** ω-NH_2 oriented towards E103 and COOH close to R169	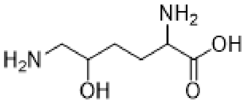 2,6-diamino-5-hydroxyhexanoic acid
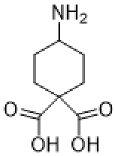 4-aminocyclohexane-1,1-dicarboxylic acid	**-4.99** NH_2 oriented towards E103 and di-COOH oriented towards a positively charged area (R162-R169)	not tested
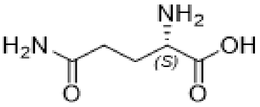 *L*-glutamine	**-4.68** NH_2 quite far but oriented towards E103 and COOH between D295 and R169	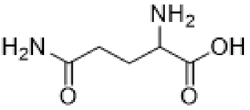 glutamine
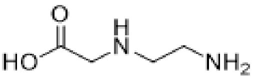 (2-aminoethyl)glycine	**-4.70** NH_2 oriented towards E103 and COOH oriented towards a positively charged area (R162-R169)	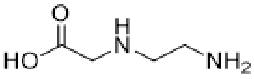 (2-aminoethyl)glycine
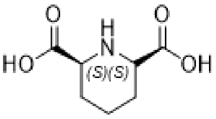 (2*S*,6*S*)-piperidine-2,6-dicarboxylic acid	**-6.57** One COOH oriented towards E103 and the other one towards a positively charged area (R162-R169)	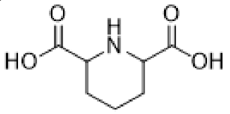 piperidine dicarboxylate

* The reported energies of binding are related to the closest assumed conformation of a product after reductive amination (or reduction for piperidine dicarboxylate).

Supplementary Table 5. Analytical yields obtained with enzymes selected for activity towards bulkier substrates octanal (**8a**), 4-phenylbutan-2-one (**9a**), together with the reference substrates cyclohexanone (**2a**) and hexanal (**7a**).

	**Transformation BL21**	**Induction**	**Lysate**	**Bradford (mg/ml)**	**Analytical yield of the corresponding amine (%)**			
					Cyclohexanone (**2a**)	Hexanal (**7a**)	Octanal (**8a**)	4-phenylbutan-2-one (**9a**)
A0A138ZYM0	+	-	+	1.79	8.5	0.1	0.0	nd
MGYP000165741795	+	-	+	2.75	68.4	0.6	0.0	nd
MGYP001184784288	+	+	+	2.75	69.1	0.5	0.1	0.1
MGYP000563817680	+	+	-	3.19	52.0	0.0	0.1	0.0
MGYP000963671166	+	+	+	3.95	15.0	0.1	0.0	nd
GUT_GENOME220312_01244	+	+	+	3.54	13.3	0.1	0.0	0.1
MGYP001470669209	+	+	-	3.30	0.2	nd	0.0	nd
METDB-02	+	+	+	2.97	69.8	0.5	0.7	3.2
METDB-03	+	+	+	1.39	58.1	1.0	4.6	14.0
GUT_GENOME000603_01215	+	+	+	3.11	18.1	0.2	0.1	nd
MGYP000417998329	+	-	-	3.72	28.8	0.1	0.1	0.1
MGYP000037226974	+	+	+	3.57	29.9	0.1	0.0	nd
MGYP000528433141	+	+	-	2.37	54.5	0.6	0.0	0.1
MGYP000288071185	+	+	+	3.91	24.7	0.2	0.0	nd
MGYP000273355962	+	+	+	3.76	52.0	0.5	0.0	nd
IGC-14	+	+	+	3.23	53.6	0.8	0.0	nd
MGYP001051592001	+	+	+	2.30	57.8	0.6	0.1	0.1
*Cfus*AmDH	+	+	+	3.63	57.3	0.3	nd	nd
MATOUAmDH2	+	+	-	3.13	nd	0.9	1.1	0.1
*Cfus*AmDH-W145A	+	+	+	1.82	nd	1.3	8.2	17.4
*Cfus*AmDH-W145A_purified	+	+	+	10.5	nd	10.2	6.9	26.7
without overexpressed enzyme	+	-	-	2.15	nd	nd	nd	nd

nd: not detected; "+" and "-" in "transformation BL21", "induction" and "lysate" indicate visible or negative band on SDS-PAGE gel respectively. Reactions conditions: 10 mM substrate, 5% DMSO (v/v), 2 M NH_4HCO_2 buffer pH 9.0, 0.2 mM $NADP^+$, 0.2 mM NAD^+, 11 mM glucose, 3 U ml^{-1} GDH-105, 20 µL crude cell lysate (0.5 mg mL^{-1} in case of purified enzyme obtained from previous published work), 24 h, 30°C. Analytical yields were deduced from calibration curves after derivatization with benzoyl chloride as described in Methods.

Supplementary Table 6. Analytical yields obtained with enzymes selected for activity towards 3C-ketone **5a** together with reference substrates **2a** and **12a**.

	Transformation BL21	Induction	Lysate	Bradford (mg/mL)	Analytical yield in corresponding amine (%) hexan-3-one (5a)	hexan-2-one (12a)	Cyclohexanone (2a)
A0A1X1SL76	+	-	+	3.29	0.2	0.3	6.0
A0A2U9PR62	+	+	+	3.54	1.5	0.4	57.0
I7GAY8	+	-	+	2.82	1.3	0.3	62.6
MGYP000173707123	+	+	-	3.22	1.1	0.4	69.0
MGYP000211951848	+	+	+	2.34	6.6	3.9	56.7
MGYP000529792485	+	+	-	1.48	1.0	0.5	4.3
MGYP000689866863	+	+	-	2.84	0.7	0.2	8.3
MGYP001209562846	+	+	-	1.93	5.4	1.9	27.1
A0A519DQZ7	+	+	+	2.23	0.4	0.4	43.9
A0A229HGK2	+	+	+	2.96	8.8	15.1	63.6
A0A1C6LG02	+	+	-	2.72	0.9	0.3	nd
A0A1Q4UXH9	+	+	+	2.67	6.1	5.7	10.5
MGYP000970333896	+	+	+	1.90	nd	nd	nd
MGYP001097556939	+	+	-	2.09	nd	nd	nd
GUT_GENOME133859_01404	+	+	+	2.10	nd	nd	nd
GUT_GENOME103710_02937	+	+	-	2.71	nd	nd	nd
MGYP001042153616	+	-	-	2.14	nd	nd	nd
A0A4S3B2N2	+	+	+	ndd	nd	nd	10.8
MGYP000075503774	+	+	+	1.71	nd	nd	1.4
MGYP000827707224	+	+	+	3.87	nd	nd	1.6
GUT_GENOME149041_01575	+	+	+	3.46	nd	nd	nd
MGYP001048001229	+	+	+	3.34	nd	nd	nd
GUT_GENOME088561_01464	+	+	+	1.93	nd	nd	1.1
MGYP000457035951	+	+	+	1.39	nd	nd	0.3
GUT_GENOME149657_00029	+	-	+	2.61	nd	nd	0.3
MGYP001082061073	+	-	+	2.01	nd	nd	0.0
A0A138ZYM0	+	-	+	1.79	0.2	nd	62.8
MGYP001470669209	+	+	-	3.30	nd	nd	0.2
A0A365ZD63	+	+	+	3.41	5.8	nd	79.8
*Porti*AmDH	+	-	-	2.94	nd	nd	nd
*Cfus*AmDH	+	+	+	3.63	nd	nd	nd
A0A646KJR1	+	+	-	3.39	2.7	nd	nd
*Micro*AmDH	+	-	-	3.28	1.7	nd	23.0
*Micro*AmDH_purified	+	+	-	4.90	9.1	nd	66.4
without overexpressed enzyme	+	-	-	2.15	nd	nd	nd

nd: not detected; ndd: not determined; "+" and "-" in "transformation BL21", "induction" and "lysate" indicate visible or negative band on SDS-PAGE gel, respectively. Reactions conditions: 10 mM substrate, 5% DMSO, 2 M NH_4HCO_2 buffer pH 9.0, 0.2 mM $NADP^+$, 0.2 mM NAD^+, 11 mM glucose, 3 U ml^{-1} GDH-105, 20 µL crude cell lysate (0.5 mg mL^{-1} in case of purified enzyme obtained from previous published work), 24 h, 30°C. Analytical yields were deduced from calibration curves after derivatization with benzoyl chloride as described in Methods.

Supplementary Table 7. Analytical yields obtained with enzymes selected for activity with cyclohexanone (**2a**) and methylamine (**c**) together with cyclohexanone (**2a**) and ammonia (**b**).

	Transformation BL21	Induction	Lysate	Bradford (mg/mL)	Analytical yield in corresponding amine (%)	
					Cyclohexanone (**2a**) + NH3 (**b**)	Cyclohexanone (**2a**) + MeNH2 (**c**)
A0A365ZD63	+	+	+	3.41	97.1	86.4
A0A2G6MY80	+	+	+	3.02	93.3	84.4
A0A2G6Q2D5	+	+	+	2.41	87.8	49.9
A0A2R3MYQ8	+	+	+	3.76	97.3	82.6
A0A3P2AE89	+	+	+	3.35	100.4	82.3
A0A4R9C3Q3	+	+	+	3.61	84.7	58.0
MGYP000142934144	+	+	+	2.66	86.9	74.9
MGYP000879705604	+	+	+	0.71	82.7	73.5
MGYP000893205724	+	+	+	2.94	91.4	79.8
MGYP000996099441	+	+	+	2.51	99.3	83.2
MGYP000619964244	+	+	+	3.23	77.5	0.5
IGC-32	+	+	-	3.35	82.9	10.4
MGYP000230715187	+	+	+	3.65	4.6	nd
A0A1A2V6I1	+	+	+	2.54	37.8	1.3
A0A1A3HJ65	+	+	-	3.36	54.3	3.1
MGYP001064279418	+	-	-	2.01	49.1	1.9
MGYP001136017405	+	-	-	2.15	1.3	nd
*Cfus*AmDH	+	+	+	3.63	67.4	1.7
*Micro*AmDH	+	+	+	3.81	27.9	0.4
*Micro*AmDH_purified	+	+	-	4.90	74.4	17.0
without overexpressed enzyme	+	-	-	2.15	nd	nd

nd: not detected; "+" and "-" in "transformation BL21", "induction" and "lysate" indicate visible or negative band on SDS-PAGE gel respectively. Reactions conditions: 10 mM cyclohexanone (**2a**), 200 mM TRIS.HCl pH 9 (for reaction with **c**) or 2 M NH_4HCO_2 buffer pH 9.0 (for reaction with **b**), 250 mM **c** (only for reaction with **c**), 0.2 mM $NADP^+$, 0.2 mM NAD^+, 11 mM glucose, 3 U ml^{-1} GDH-105, 20 µL crude cell lysate ((0.5 mg mL^{-1} in case of purified enzyme obtained from previous published work), 24 h, 30°C. Analytical yields were deduced from calibration curves and after derivatization with benzoyl chloride as described in Methods.

Supplementary Table 8. Energies of binding obtained from docking experiments of *N*-methylcyclohexyliminium and *N*-ethylcyclohexyliminium in A0A365ZD63, MGYP000996099441, MGYP000893205724, A0A4R9C3Q3, IGC-32 and A0A2G6MY80.

	***N*-methylcyclohexyliminium**	***N*-ethylcyclohexyliminium**
A0A365ZD63	-7.56	-7.68
MGYP000996099441	-7.49	-7.78
MGYP000893205724	-7.32	-7.53
A0A4R9C3Q3	-7.27	-7.61
IGC-32	-5.80	-6.03
A0A2G6MY80	-7.11	-7.81

All the 10 top conformations displayed the same energies of binding for each docked molecule/enzyme couple. Energies of binding are expressed in kcal mol^{-1}. We can note that the extremely low analytical yields with ethylamine (**d**) were not correlated with energies of binding or conformations calculated with docking of the *N*-ethylcyclohexyliminium, which were quite similar or even better to *N*-methylcyclohexyliminium ones.

Supplementary Table 9. Number of NAD(P)-dependent enzyme sequences collected through our two-step protocol.

Database	Number of sequences
MGnify	10,244,362
TrEMBL	4,124,305
GEM	3,037,031
Human-gut	1,617,023
OM-RGC	639,656
MATOUv2	364,087
MetDB	123,990
IGC	97,083
SMAGs	56,585
SwissProt	11,623
Total	*20,315,745*

Supplementary Table 10. Number of AmDH sequences collected through our three-step protocol. (A) : with all_ASMC_no_nad, (B): with all_ASMC_no_nad and score ≥ 100, (C): keep sequences between 250 and 500 amino acids in length, (D): with all_ASMC_nad_dom and score ≥ 50

Database	*A*	*B*	*C*	*D*
MGnify	27,386	17,575	10,744	10,331
TrEMBL	6,898	6,536	6,393	8,190*
Human-gut	5,944	5,840	5,704	5,700
GEM	3,069	2,612	2,612	2,610
IGC	401	340	295	293
OM-RGC	353	215	148	143
MetDB	27	11	8	8
MATOUv2	94	25	10	6
SMAGs	1	1	1	1
Total				*27,282*

*includes the 1,816 nat-AmDHs used to build the HMM profiles all_ASMC_no_nad and all_ASMC_nad_dom.

Supplementary Table 11. Screening results of the 301 HMM profiles from the SSF51735 entry against the UniProtKB resource (SwissProt+TrEMBL). The selected cut-off score is written in bold. TP: True Positive, FP: False Positive, FN: False Negative, TN: True Negative, sens: sensitivity, spec: specificity.

cut-off	TP	FP	FN	TN	sens	spec	1-spec
SwissProt (total number of hits: 20,102)							
0	11,983	8,119	0	0	1	0	1
**50**	**11,807**	**1,031**	**176**	**7,088**	**0.99**	**0.87**	**0.13**
100	10,633	31	1,350	8,088	0.89	1	0
150	6,411	0	5,572	8,119	0.54	1	0
200	2,467	0	9,516	8,119	0.21	1	0
250	1,035	0	10,948	8,119	0.09	1	0
300	292	0	11,691	8,119	0.02	1	0
350	29	0	11,954	8,119	0	1	0
TrEMBL (total number of hits: 6,158,043)							
0	4,311,774	1,846,269	0	0	1	0	1
**50**	**4,163,780**	**504,816**	**147,994**	**1,341,453**	**0.97**	**0.73**	**0.27**
100	3,615,679	103,173	696,095	1,743,096	0.84	0.94	0.06
150	2,323,894	48,517	1,987,880	1,797,752	0.54	0.97	0.03
200	1,170,805	23,205	3,140,969	1,823,064	0.27	0.99	0.01
250	518,411	10,097	3,793,363	1,836,172	0.12	0.99	0.01
300	114,121	2,512	4,197,653	1,843,757	0.03	1	0
350	2,899	165	4,308,875	1,846,104	0	1	0

Supplementary Figures

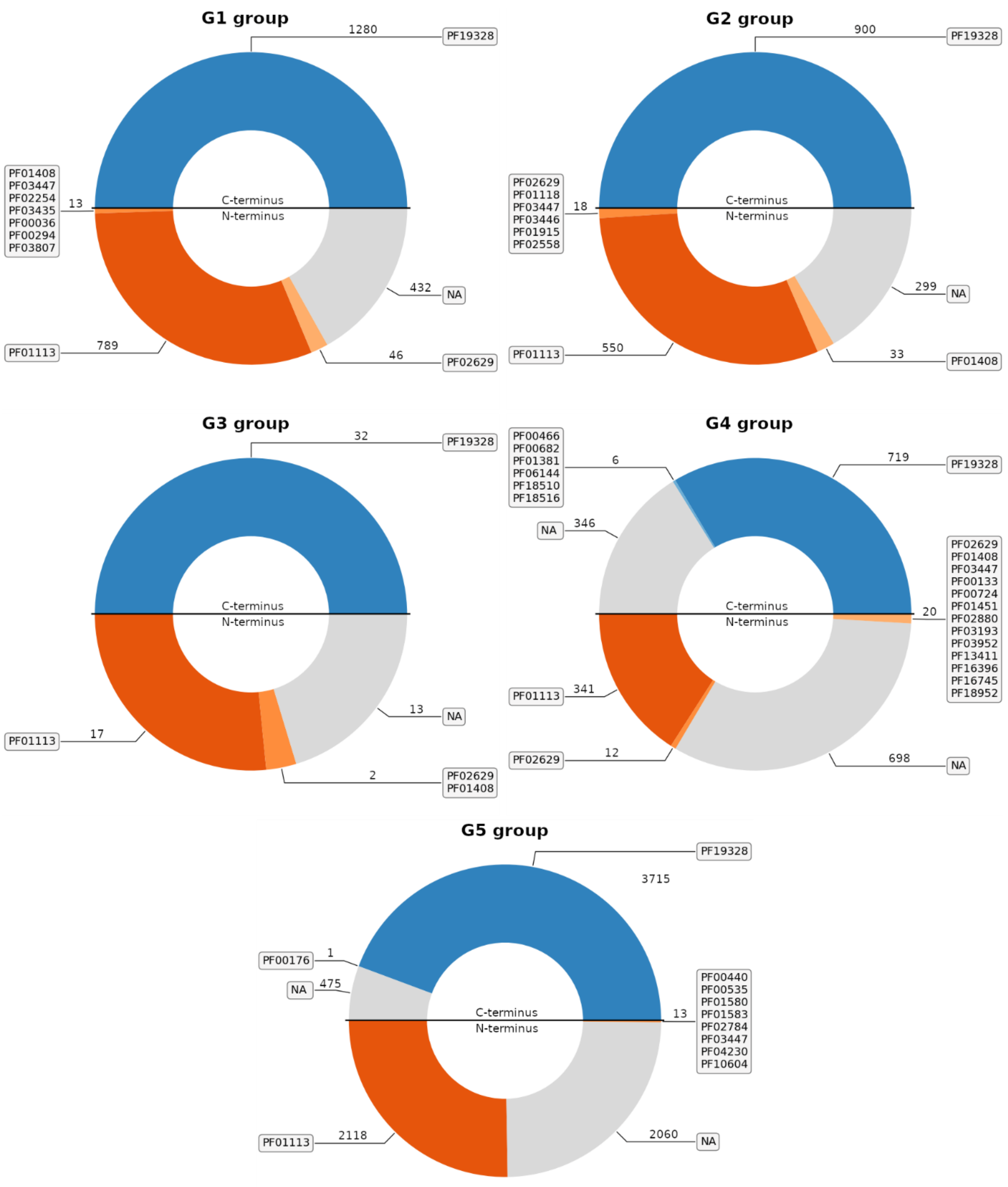

Supplementary Figure 1. Pfam annotations (version 35.0) for each ASMC group in the ref-AmDH family. Each one was built from the phylogenetic tree depicted in Figure 2B (enzyme count: 1,280 G1', 900 G2', 32 G3', 1,071 G4' and 4,191 G5'). The main annotations for the N- and C-terminus are PF01113 and PF19328, respectively ('NA' stands for 'Non annotated').

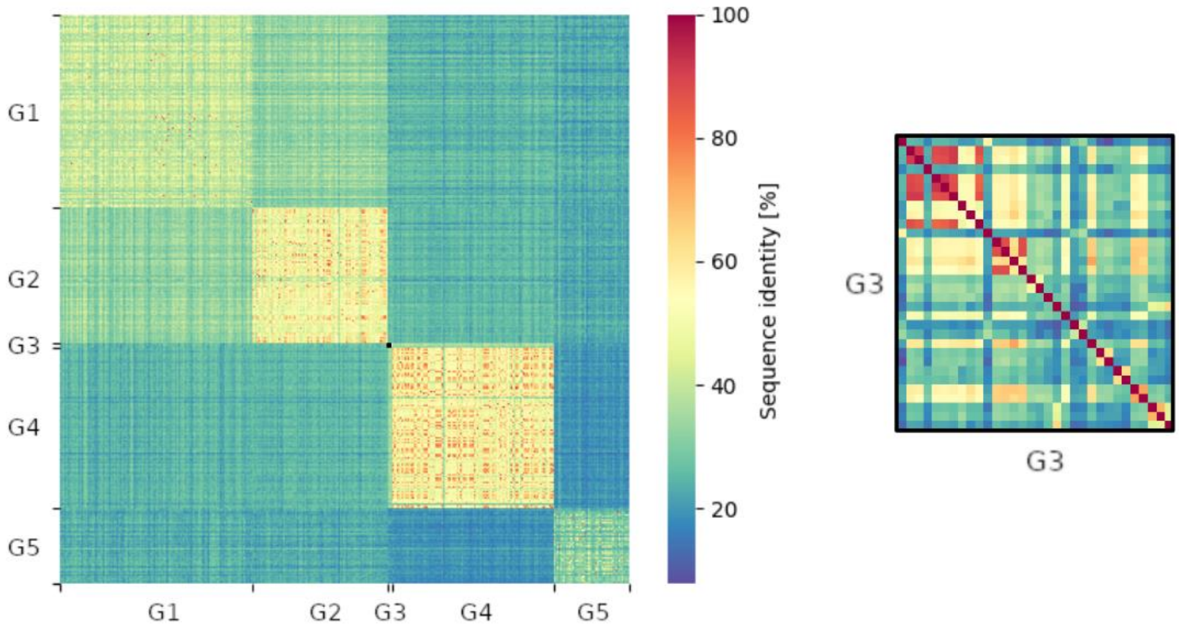

Supplementary Figure 2. Amino-acid sequence identity matrix between G1-G5 groups. G2, G3 and G4 groups include more conserved sequences (50 to 90%) than in the G1 and G5 groups, which bring together more diverse sequences (30 to 50%). Overall, the groups are separated from each other by 20-50% identity, underlining the great diversity of homologs distributed within the family. For greater clarity, the G3 group (black square) is enlarged on the right and only 500 out of 4,191 members of the G5 group were randomly selected. This matrix was designed by submitting a set of 3,783 ref-AmDH enzymes to the Clustal Omega web server[1] (details: 1,280 G1; 900 G2; 32 G3; 1,071 G4 and 500 G5).

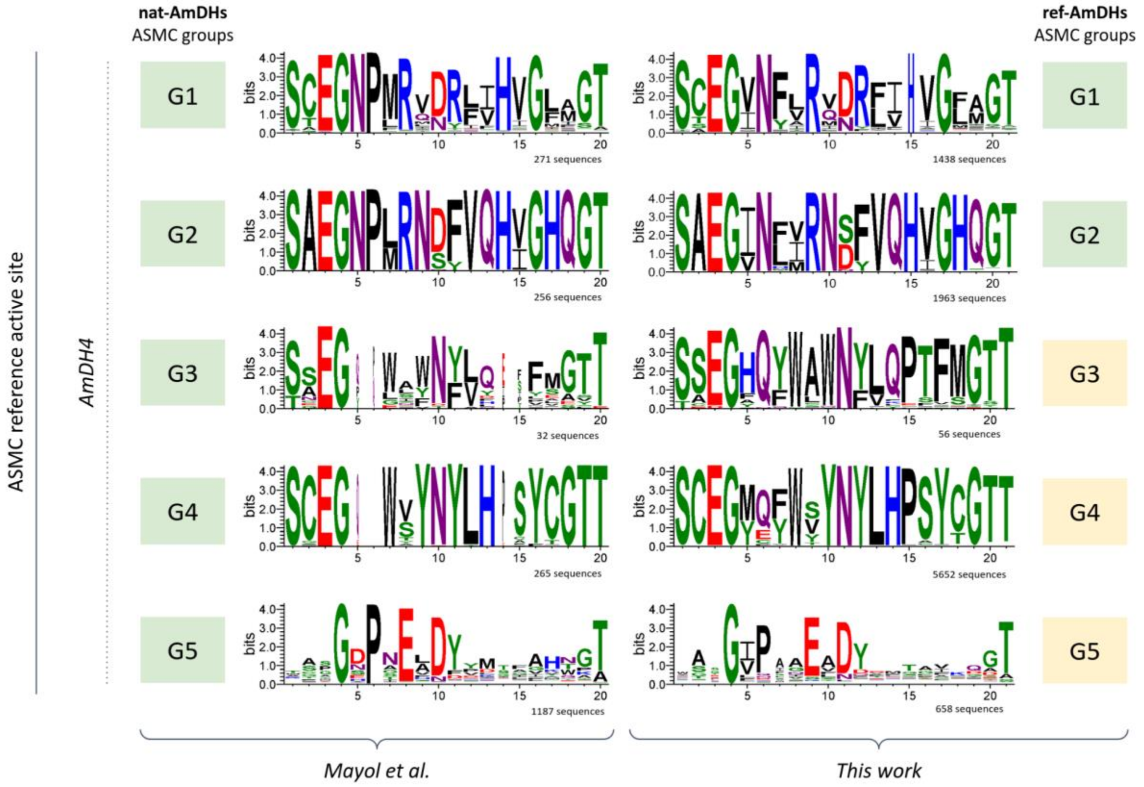

Supplementary Figure 3. Comparison of sequence logos between the former ASMC groups (left) and the new ones (right). One may note that the active site of G3 and G4 groups is better described, namely sequence logos are complete, using *Cfus*AmDH (G4 enzyme) rather than AmDH4 (G2 enzyme), as previously done.

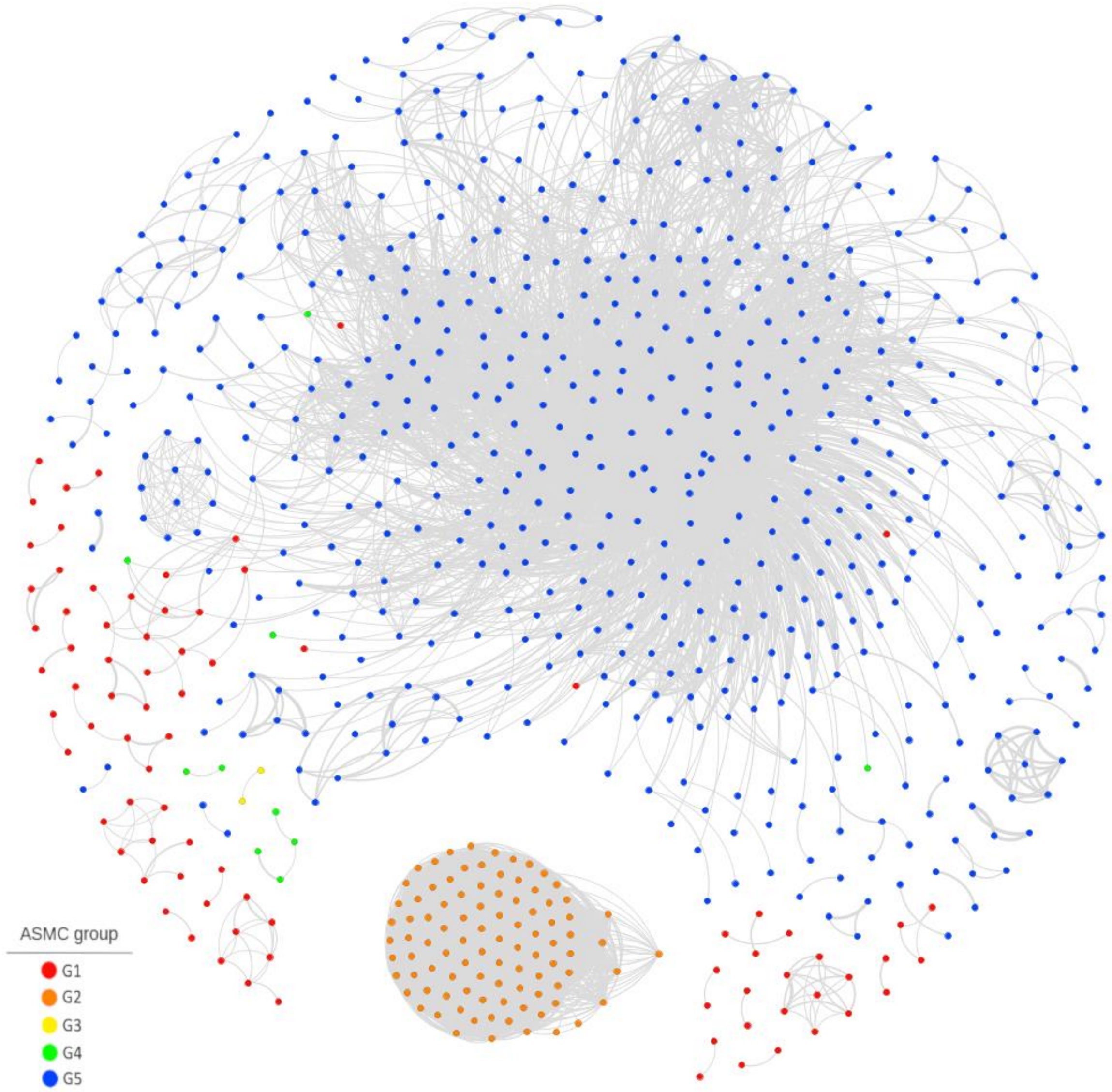

Supplementary Figure 4. NetSyn analysis of the ref-AmDH family. Each node corresponds to a protein and two proteins are linked only if a significant conserved genomic context is detected between them[2]. No consensual genomic context was found in the different groups of the ref-AmDH family, except for the G2 group (orange). Indeed, its highly-connected and dense network contains proteins annotated as “2,4-diaminopentanoate dehydrogenase” and their common genomic context includes enzymes involved in the ornithine degradation pathway. Image made with Gephi (v0.10.1) under Fruchterman-Reingold spatialisation algorithm (area: 10,000; gravity: 10; speed: 1).

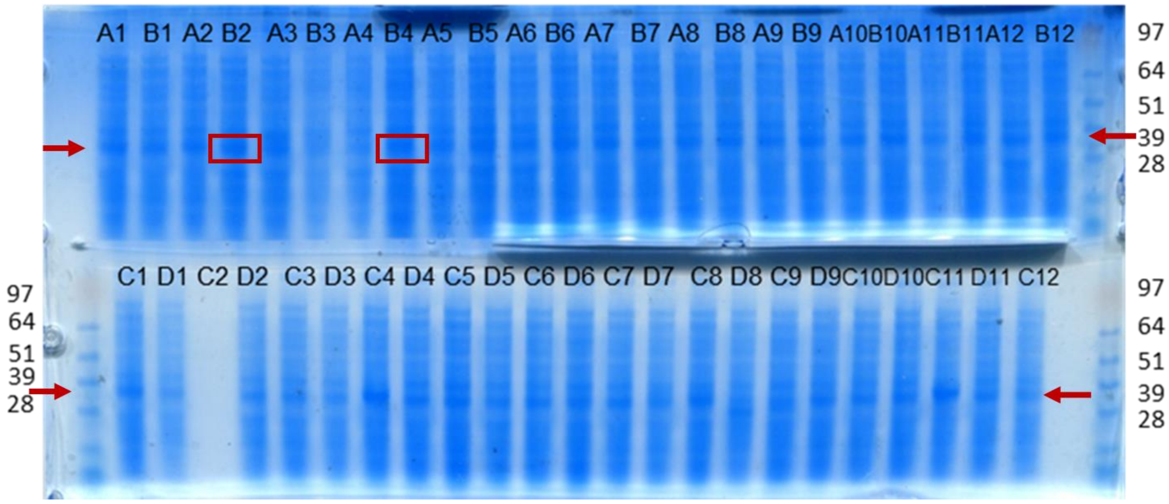

Supplementary Figure 5. SDS gels of crude cell lysates of selected AmDHs from G1 group. SDS gels using the Nu-PAGE system (Invitrogen) of crude cell lysates (10 µl deposited). The labels refer to the position in the 96-microwell plate, see Supplementary Data 2 for correspondence with Protein ID. Red arrows indicated the position of the expected overexpressed proteins. Only two protein bands were framed for clarity.

A

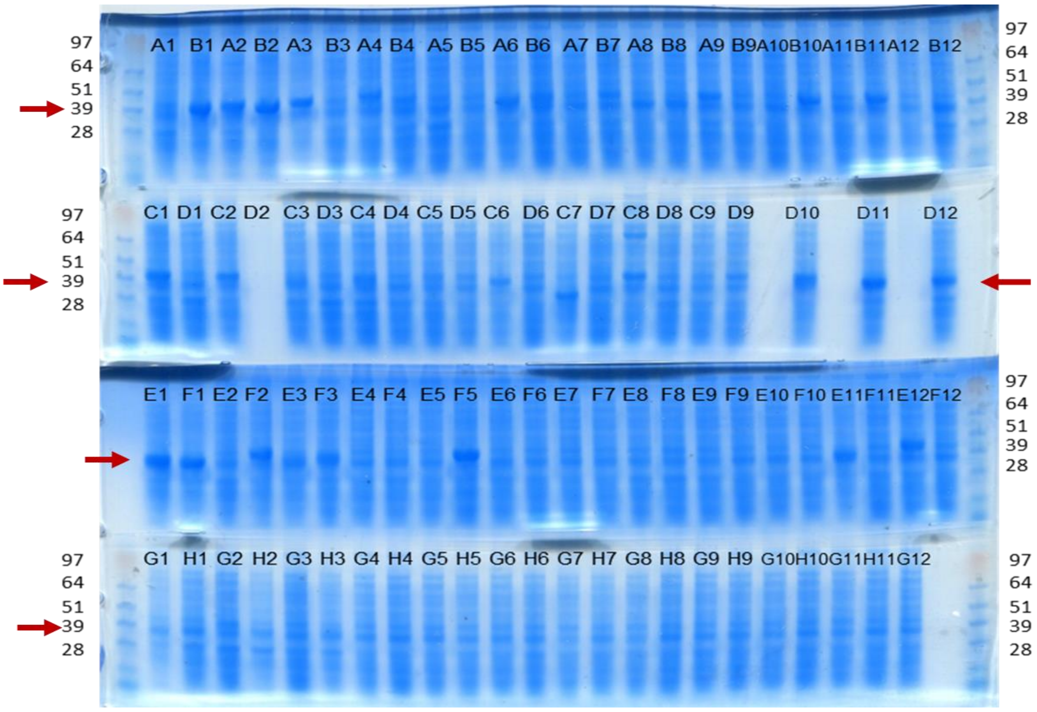

B

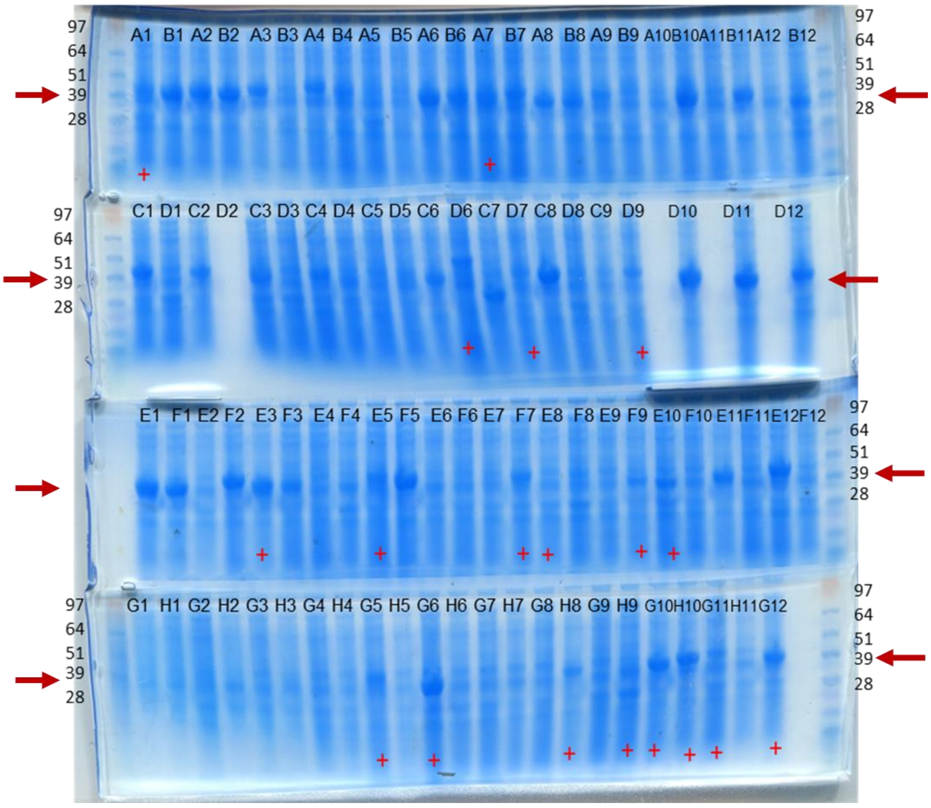

Supplementary Figure 6. SDS gels of crude cell lysates of selected AmDHs from G2-G5 groups. SDS gels using the Nu-PAGE system (Invitrogen) of crude cell lysates (10 µl deposited) obtained from induction with A) 0.5 mM IPTG and B) 1 mM IPTG. The red crosses indicate enzymes better overexpressed in condition B (50 *vs* 32). Red arrows indicated the position of the expected overexpressed proteins.The labels refer to the position in the 96-microwell plate, see Supplementary Data 3-4 for correspondence with Protein ID.

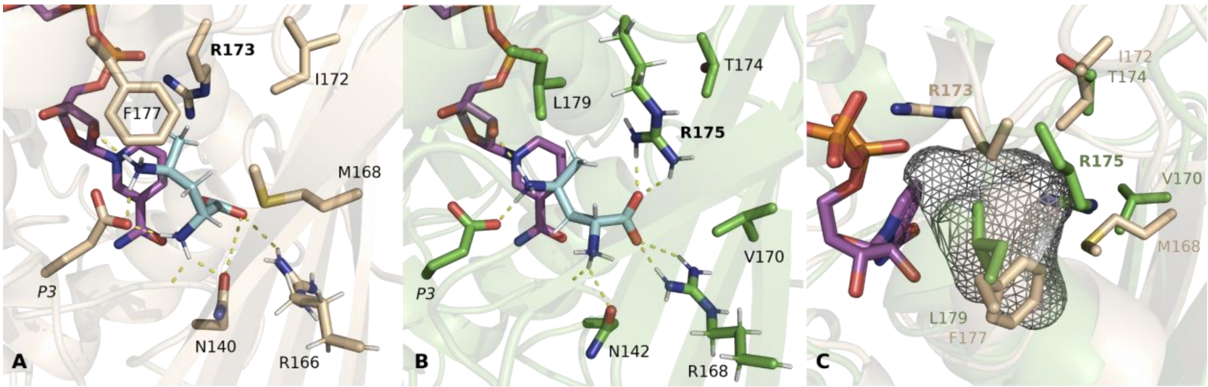

Supplementary Figure 7. PyMOL visualization of A) MGYP001132756558 (M-6558) and B) A0A540X1D9 (X1D9) active sites with docked ligands (2*R*,4*S*)-DAP (**1b**) (light blue) and nicotinamide moiety (purple). The putative conformation of bold labeled P12 arginines (R173, R175) allowed the ligand to bind quite correctly to the active site, depicting their observed activity on (2*R*,4*S*)-DAP. Hydrogen bonds are represented using yellow dotted lines. C) Top view of the superimposition of M-6558 and X1D9 active sites highlights the steric hindrance of the M-6558 cavity (meshed representation) due to residues M168, I172 and F177, which prevent R173 (M-6558) from being part of the active site in the same way as R175 (X1D9). It should be noted that, as no G1 group structure has yet been solved, G1 models based on existing X-ray structures can be misleading about the conformation and potential interactions of the P12 arginine in the active site. Two different rotamers of the P12 arginine were observed.

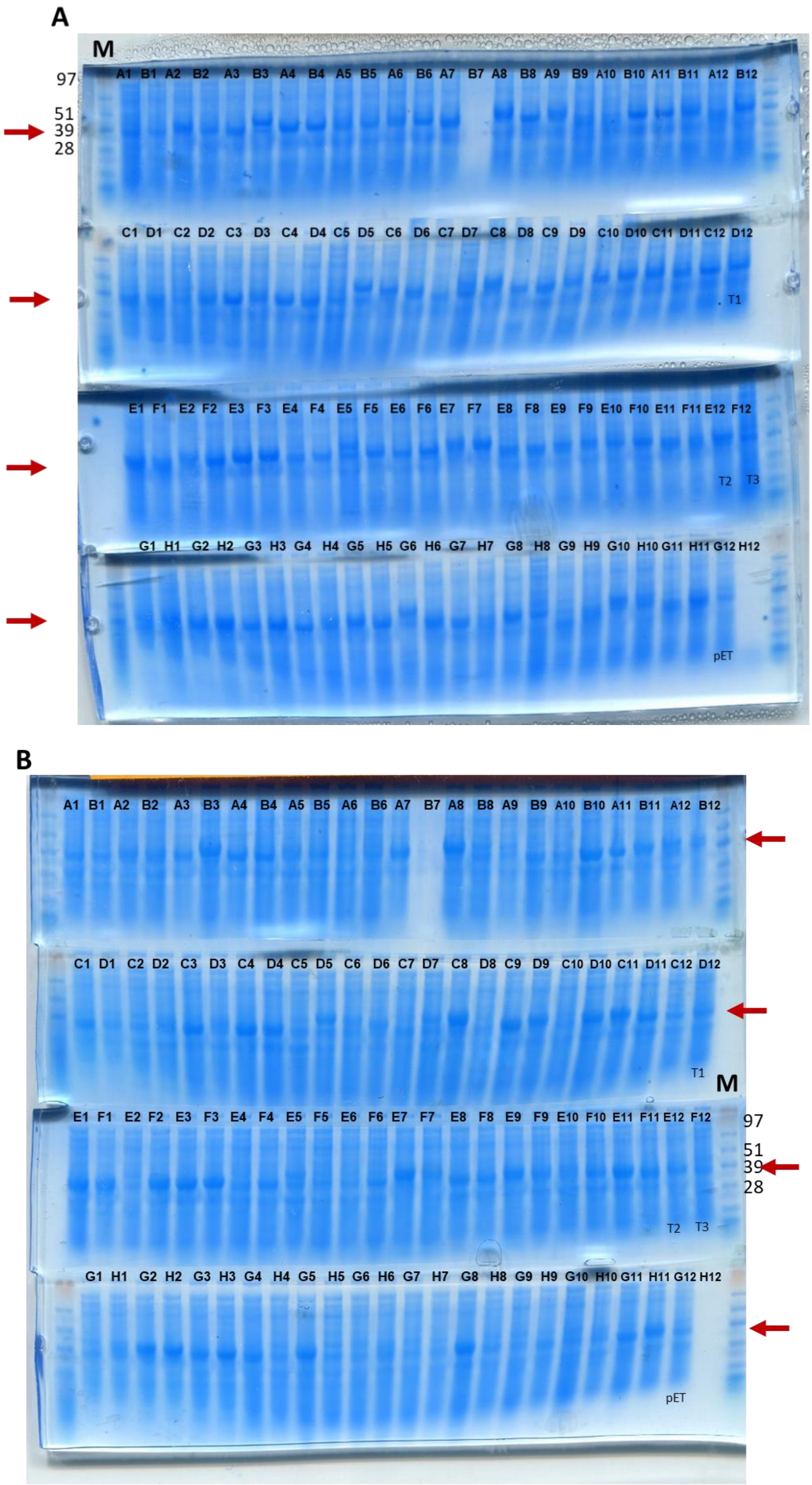

Supplementary Figure 8. SDS gels using the Nu-PAGE system (Invitrogen) of crude cell lysates (10 µl deposited) of AmDHs selected for particular features. A) Induction ; B) crude-cell lysate. M = molecular marker SeeBlue® Plus2 pre-strained standard (5 µl deposited). Red arrows indicated the position of the expected overexpressed proteins. For enzymes further purified, see Supplementary Data 7 for correspondence of position in this 96-well plate with Protein ID.

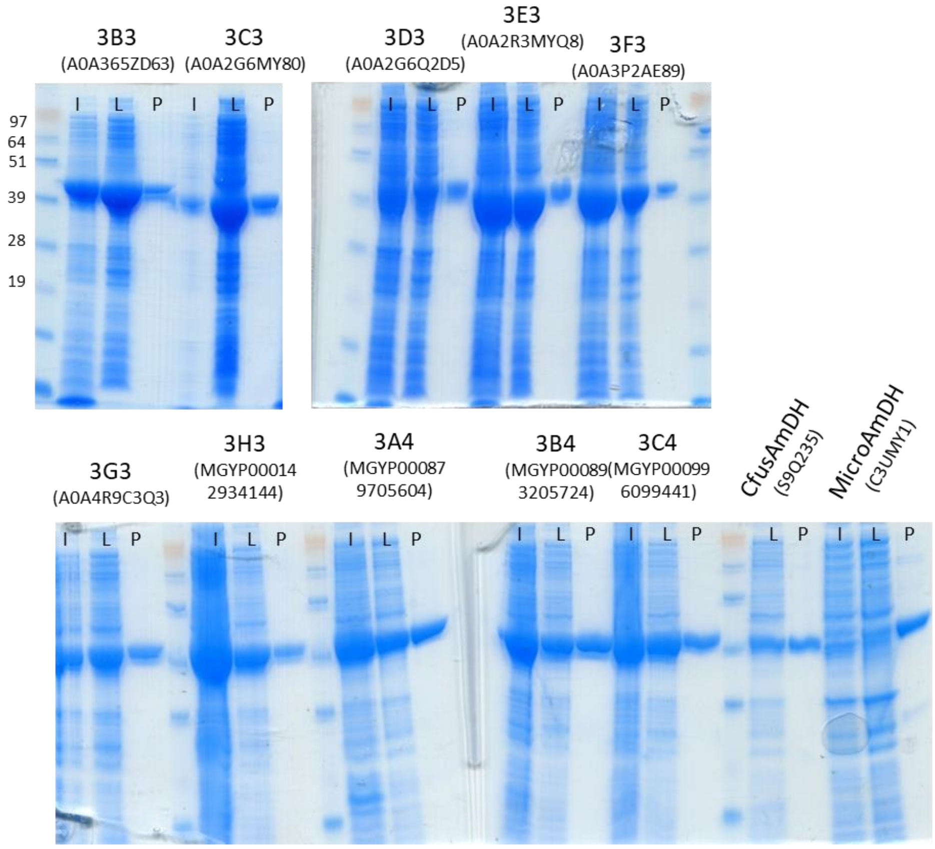

Supplementary Figure 9. SDS gels using the Nu-PAGE system (Invitrogen) of purified AmDHs, selected for amine substrate scope, obtained with Ni-NTA column QIAGEN. M = molecular marker SeeBlue® Plus2 pre-strained standard (5 µg deposited), I = induction; L = cell-free extracts (15 µg deposited) and P = purified enzymes (5 µg deposited).

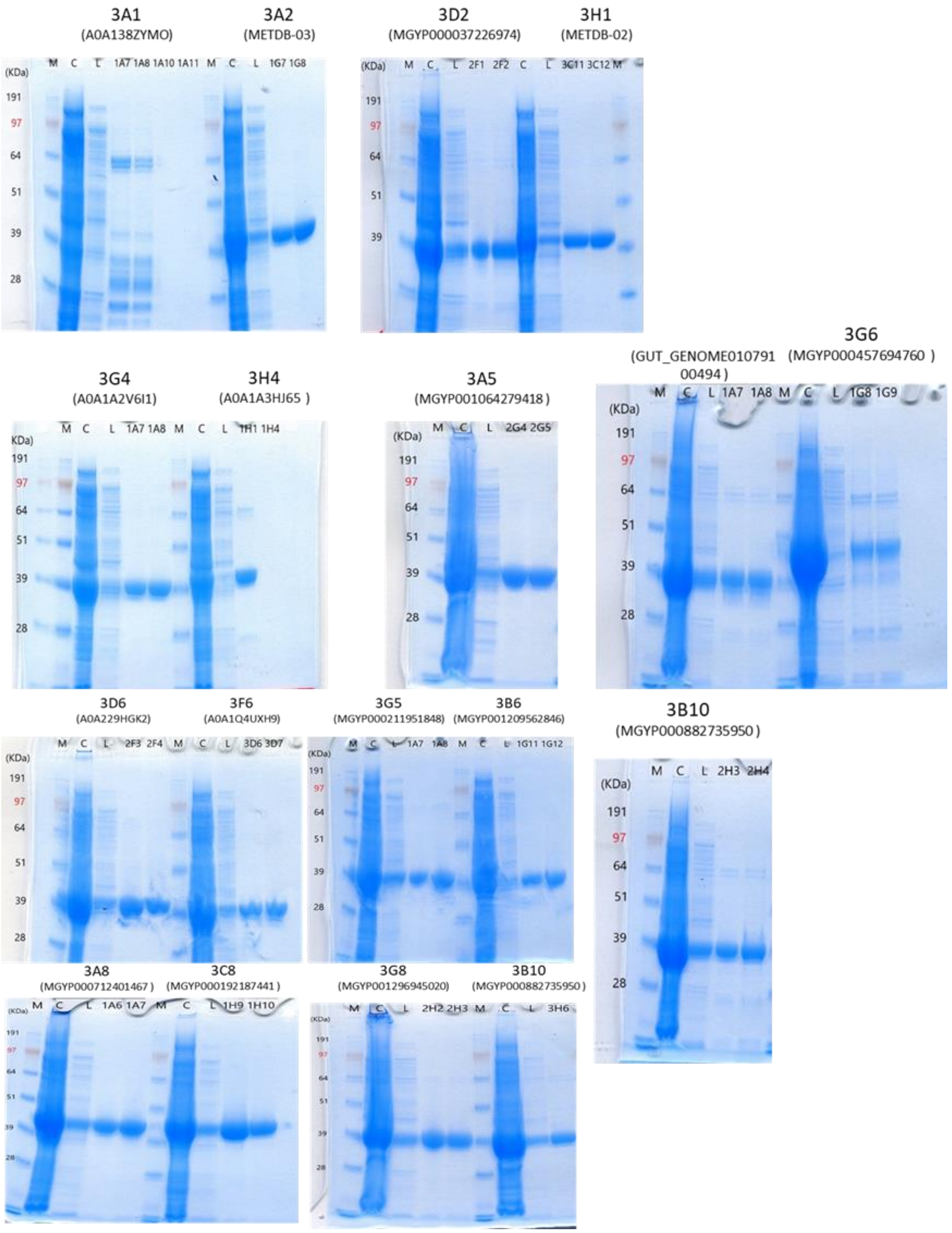

Supplementary Figure 10. SDS gels using the Nu-PAGE system (Invitrogen) of purified AmDHs, selected for the study of carbonyl substrate scope, obtained by purification in tandem with gel filtration. M = molecular marker SeeBlue® Plus2 pre-strained standard (5 µg deposited), C = insoluble part; L = cell-free extracts (15 µg deposited) and P = purified enzymes (5 µg deposited).

A

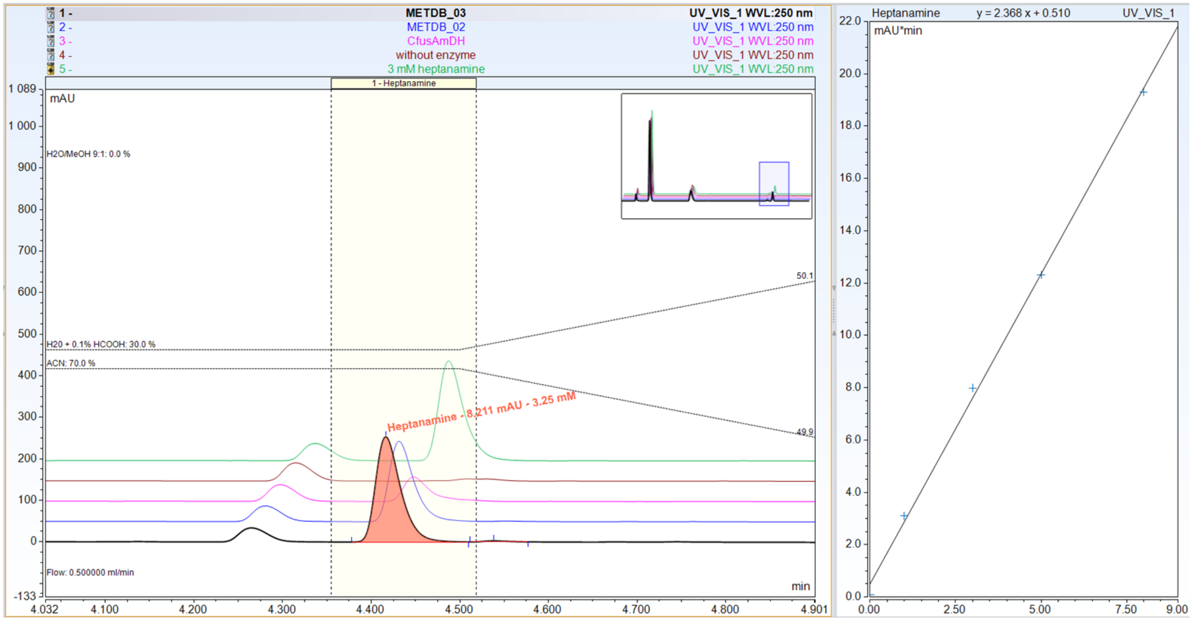
1 - METDB_03 UV_VIS_1 WVL:250 nm
2 - METDB_02 UV_VIS_1 WVL:250 nm
3 - CfusAmDH UV_VIS_1 WVL:250 nm
4 - without enzyme UV_VIS_1 WVL:250 nm
5 - 3 mM heptanamine UV_VIS_1 WVL:250 nm
1 - Heptanamine
mAU
H2O/MeOH 9:1: 0.0 %
H20 + 0.1% HCOOH: 30.0 %
ACN: 70.0 %
Heptanamine - 8.211 mAU - 3.25 mM
Flow: 0.500000 ml/min
min
Heptanamine y = 2.368 x + 0.510 UV_VIS_1
mAU*min

B

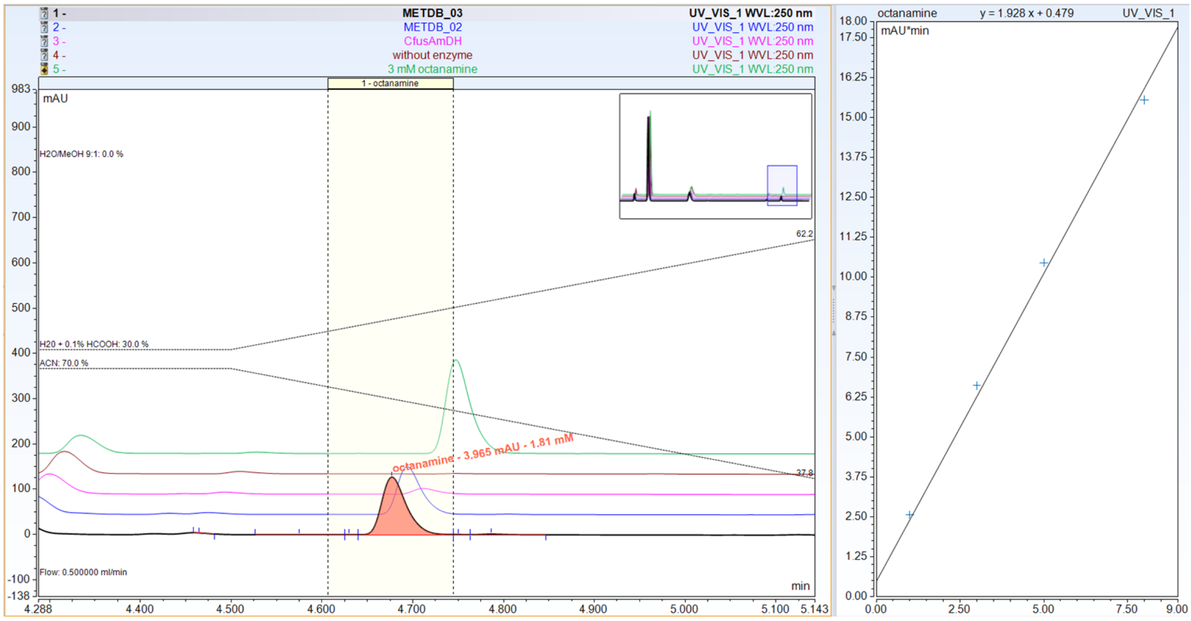
1 - METDB_03 UV_VIS_1 WVL:250 nm
2 - METDB_02 UV_VIS_1 WVL:250 nm
3 - CfusAmDH UV_VIS_1 WVL:250 nm
4 - without enzyme UV_VIS_1 WVL:250 nm
5 - 3 mM octanamine UV_VIS_1 WVL:250 nm
1 - octanamine
mAU
H2O/MeOH 9:1: 0.0 %
H20 + 0.1% HCOOH: 30.0 %
ACN: 70.0 %
octanamine - 3.965 mAU - 1.81 mM
Flow: 0.500000 ml/min
min
octanamine y = 1.928 x + 0.479 UV_VIS_1
mAU*min

C

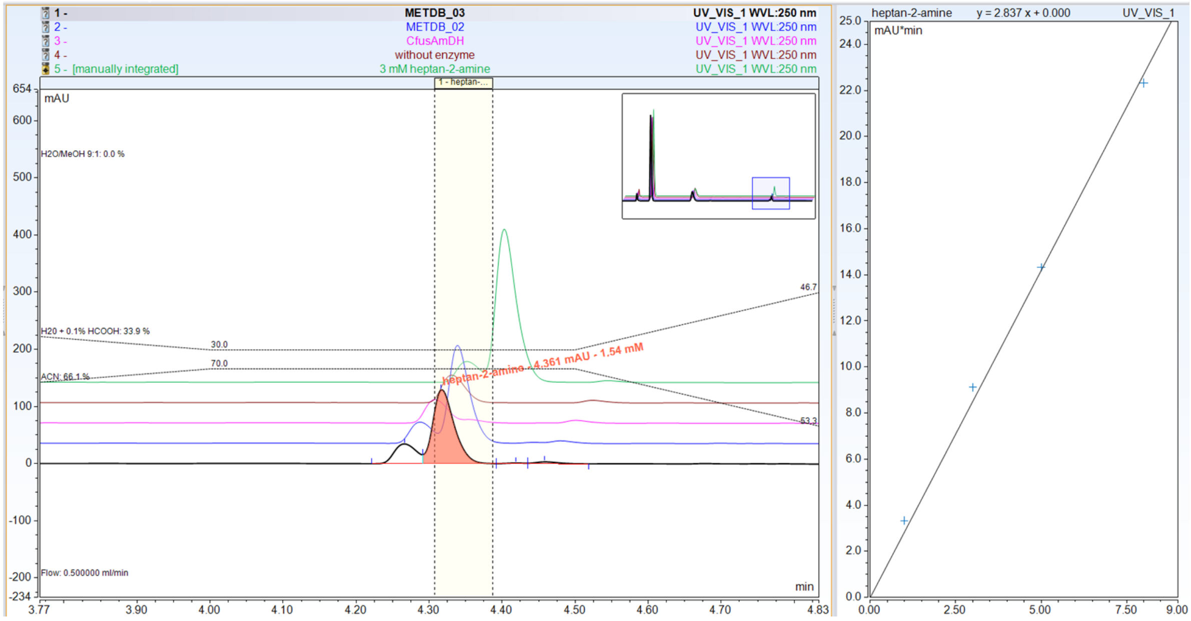

D

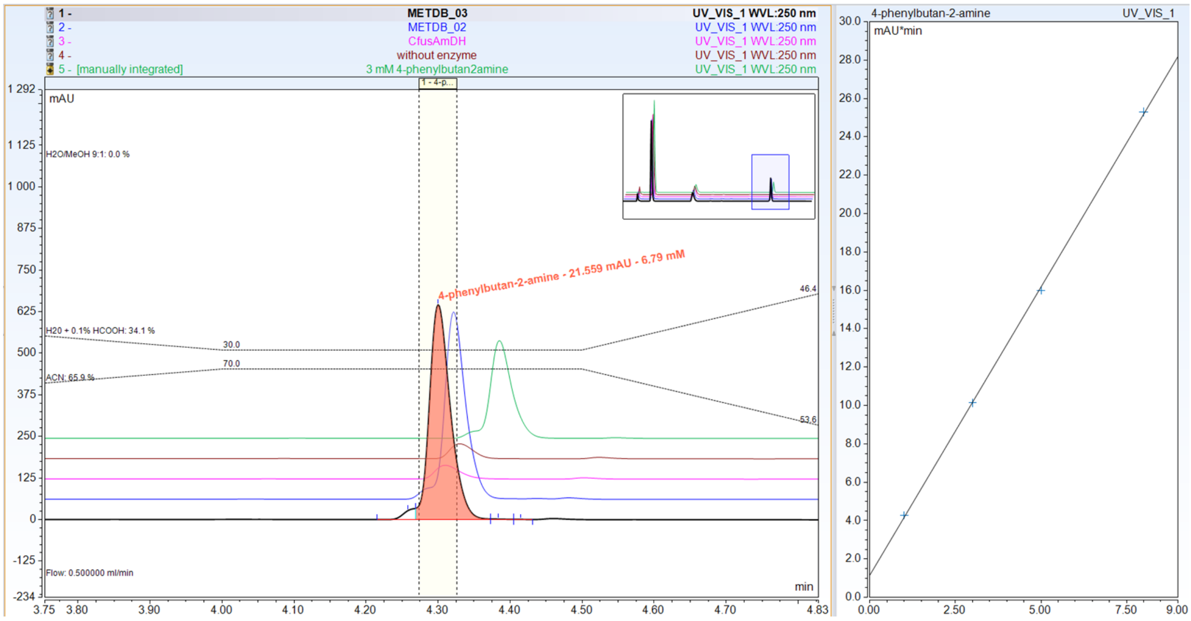

Supplementary Figure 11. UV-chromatograms (250 nm) of benzoyl-derivatized enzymatic reaction mixtures with purified METDB_03 (black), METDB_02 (blue), *Cfus*AmDH (pink), blank reaction mixture without enzyme (brown) and one calibration point (green) of amine for each corresponding tested ketones: (A) heptanal (**10a**), (B) octanal (**8a**), (C) heptan-2-one (**11a**), (D) 4-phenylbutan-2-one (**9a**). The amount of each amine was deduced from the calibration curves provided.

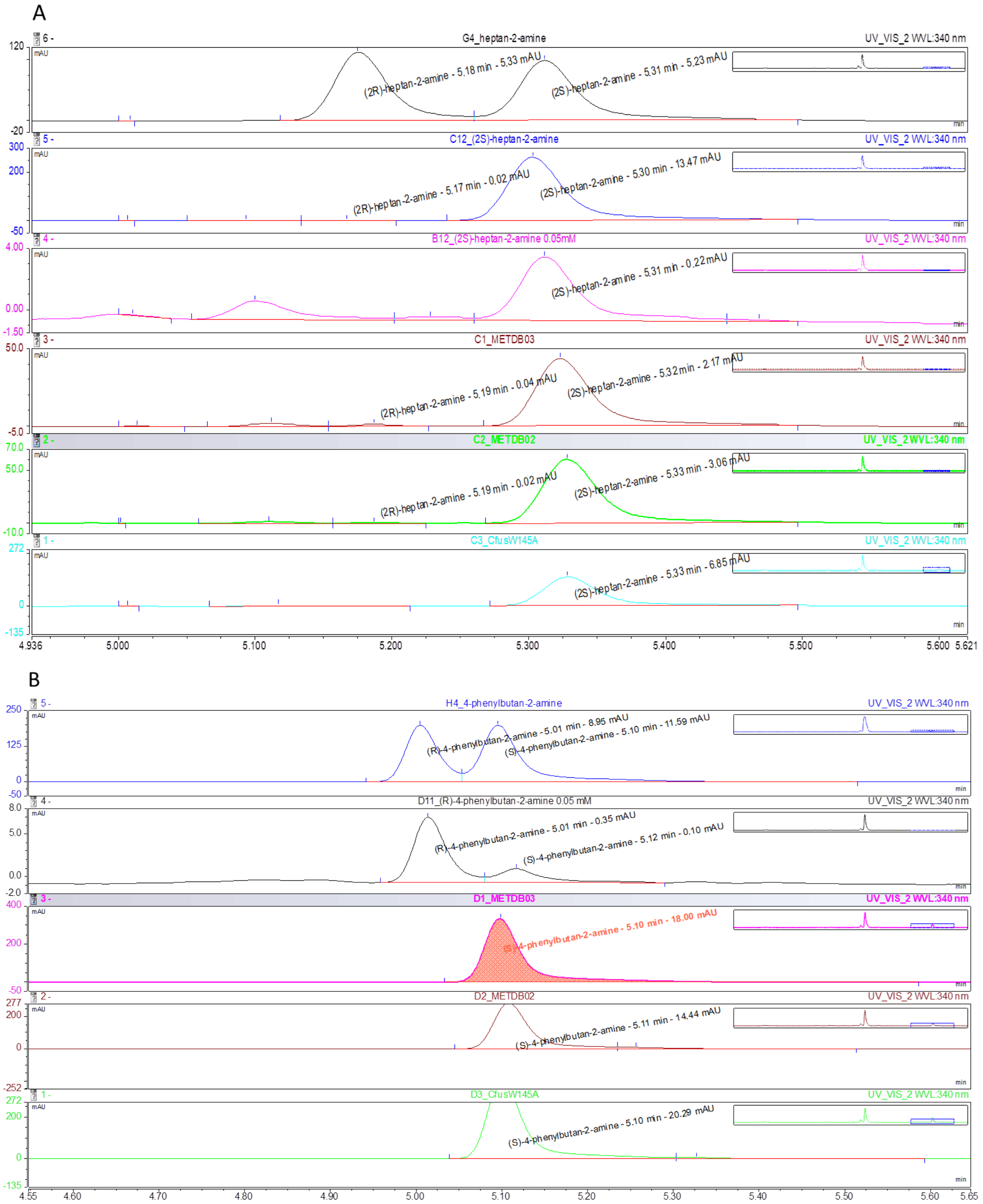

Supplementary Figure 12. UV-chromatograms (340 nm) of FDAA-derivatized enzymatic reaction mixtures with purified METDB_03, METDB_02, *Cfus*AmDH-W145A, racemic amine **9b** and **11b** and commercial enantiomerically enriched amine for reaction with (A) heptan-2-one (**11a**) and (B) 4-phenylbutan-2-one (**9a**).

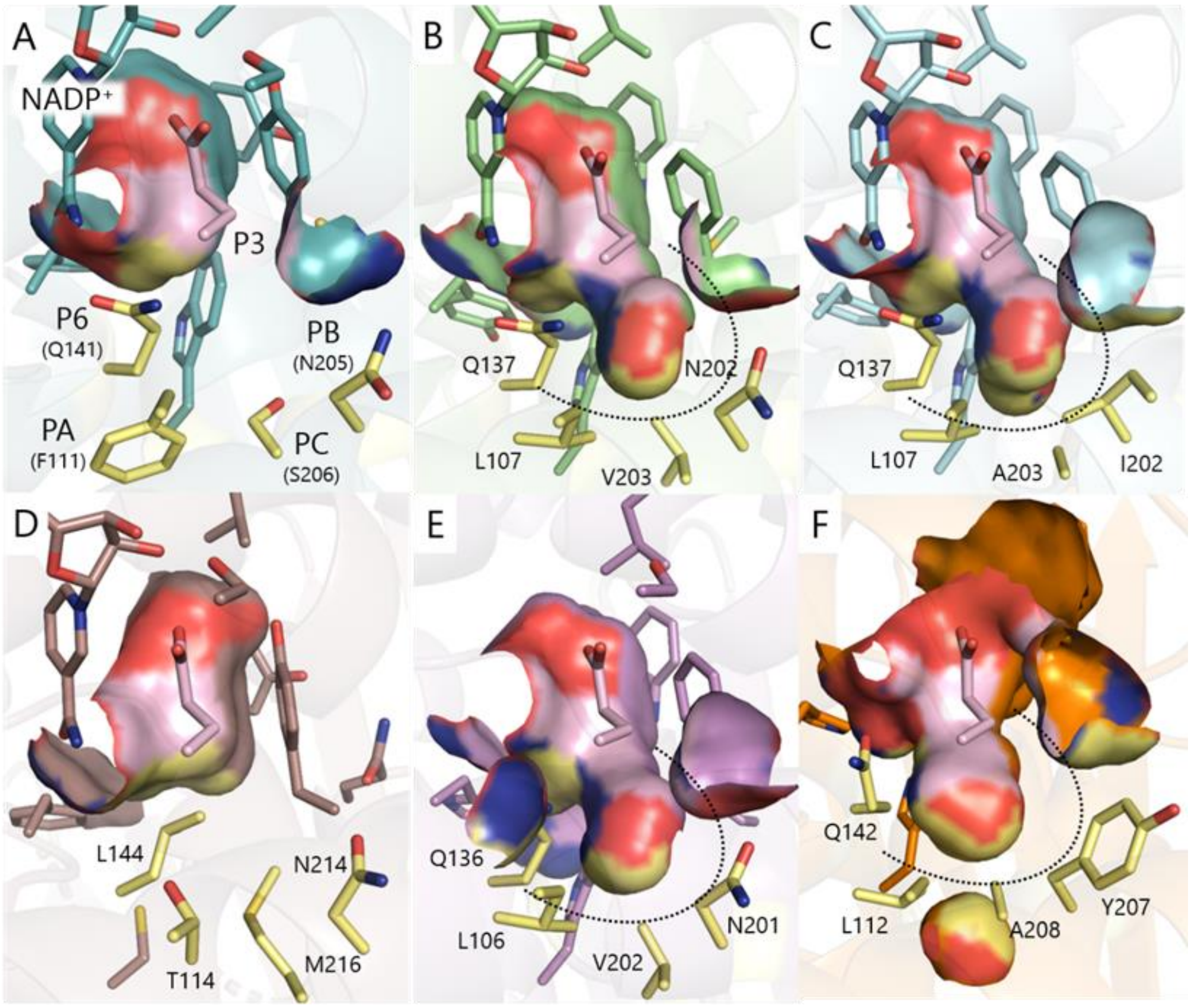

Supplementary Figure 13. Second pocket in nat-AmDHs.

This pocket is located under the catalytic residue P3 and is surrounded by residues L107 (PA), Q137 (P6), N202 (PB) and V203 (PC) in *Msme*AmDH. Thus, the selection considered enzymes with at least one small residue at PA, P6, PB or PC and also included the P1-P21 closest homologs of *Msme*AmDH, *Micro*AmDH, *Porti*AmDH and A0A646KJR1. (A) *Cfus*AmDH (PDB: 6IAU) (B) *Msme*AmDH (PDB: 6IAQ) (C) *Micro*AmDH (Model generated using Swiss Model and 6IAQ-chainA as template) (D) MATOUAmDH2 (PDB: 7R09) (E) *Porti*AmDH (Model generated using Swiss Model and 6IAQ-chainA as template) and (F) A0A646KJR1 (ColabFold model). P3 is colored pink. PA, P6, PB and PC are highlighted in yellow. The pocket is represented with the surface mode on PA, PB, PC, P3, P5, P6, P8 and P13. RMSD (*Msme*AmDH-*Cfus*AmDH) = 0.987 Å; RMSD (*Micro*AmDH-*Cfus*AmDH) = 0.878 Å; RMSD (MATOUAmDH2-*Cfus*AmDH) = 1.376 Å; RMSD (*Porti*AmDH-*Cfus*AmDH) = 0.960 Å; RMSD (*Porti*AmDH-*Cfus*AmDH) = 1.012 Å.

It is worth to note that these initial targeted positions PA, P6, PB or PC are not those which make it possible to explain the conversions of these 3C-ketones (see main text).

A

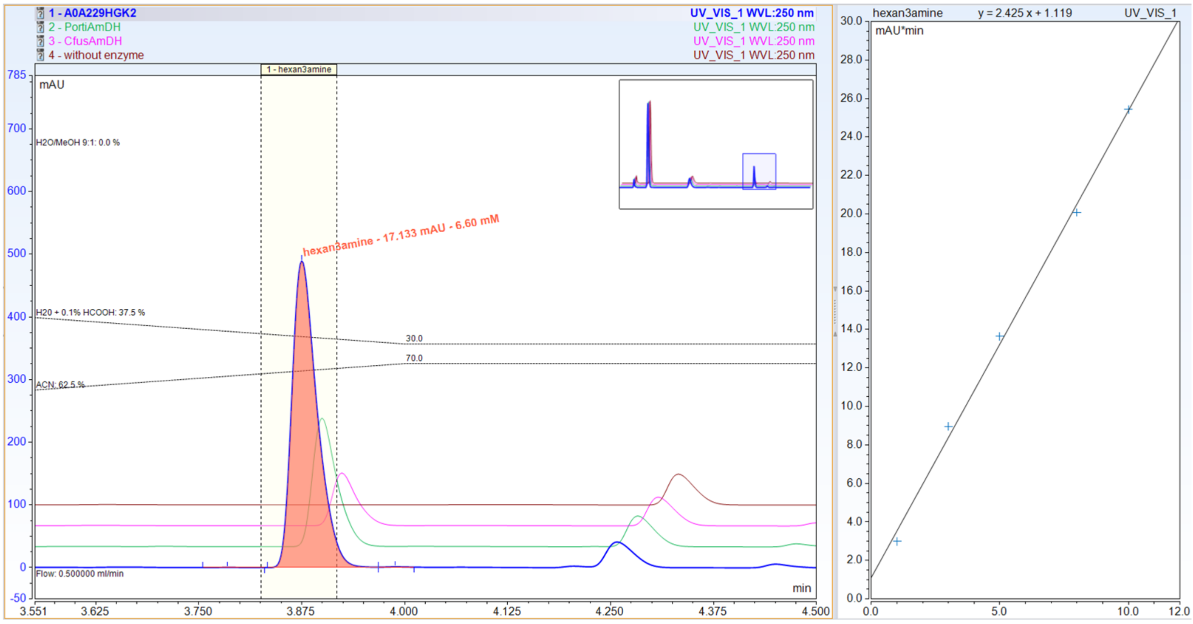

1 - A0A229HGK2
2 - PortiAmDH
3 - CfusAmDH
4 - without enzyme
UV_VIS_1 WVL:250 nm
1 - hexan3amine
mAU
hexan3amine - 17.133 mAU - 6.60 mM
H2O/MeOH 9:1: 0.0 %
H20 + 0.1% HCOOH: 37.5 %
ACN: 62.5 %
Flow: 0.500000 ml/min
min
hexan3amine
y = 2.425 x + 1.119
UV_VIS_1
mAU*min

B

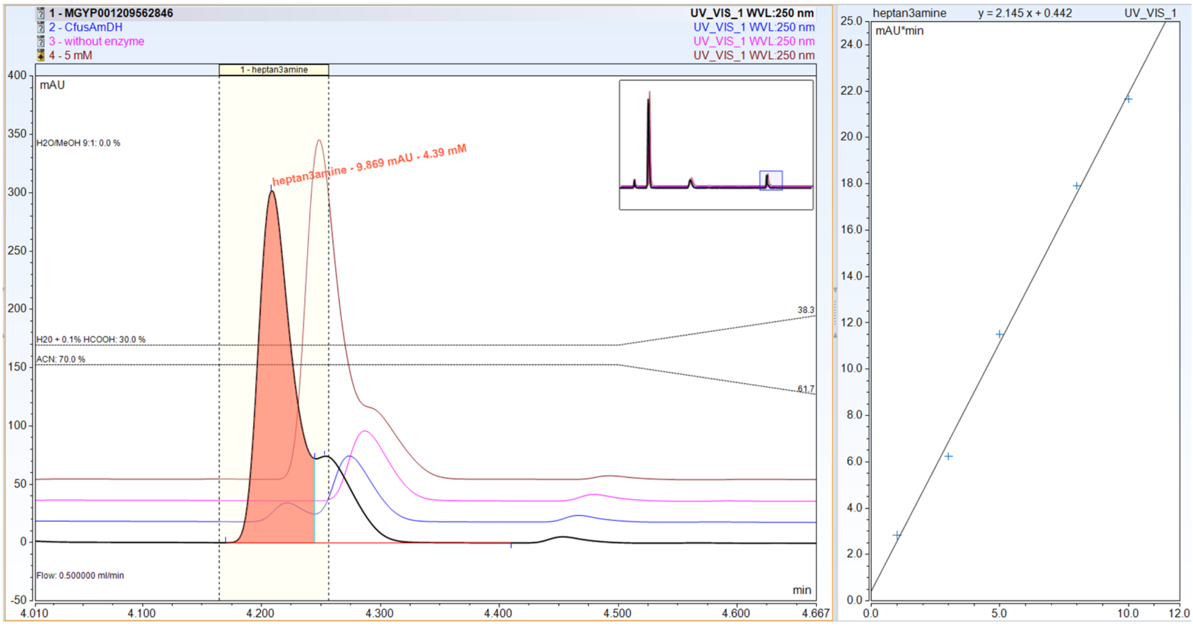

1 - MGYP001209562846
2 - CfusAmDH
3 - without enzyme
4 - 5 mM
UV_VIS_1 WVL:250 nm
1 - heptan3amine
mAU
heptan3amine - 9.869 mAU - 4.39 mM
H2O/MeOH 9:1: 0.0 %
H20 + 0.1% HCOOH: 30.0 %
ACN: 70.0 %
Flow: 0.500000 ml/min
min
heptan3amine
y = 2.145 x + 0.442
UV_VIS_1
mAU*min

C

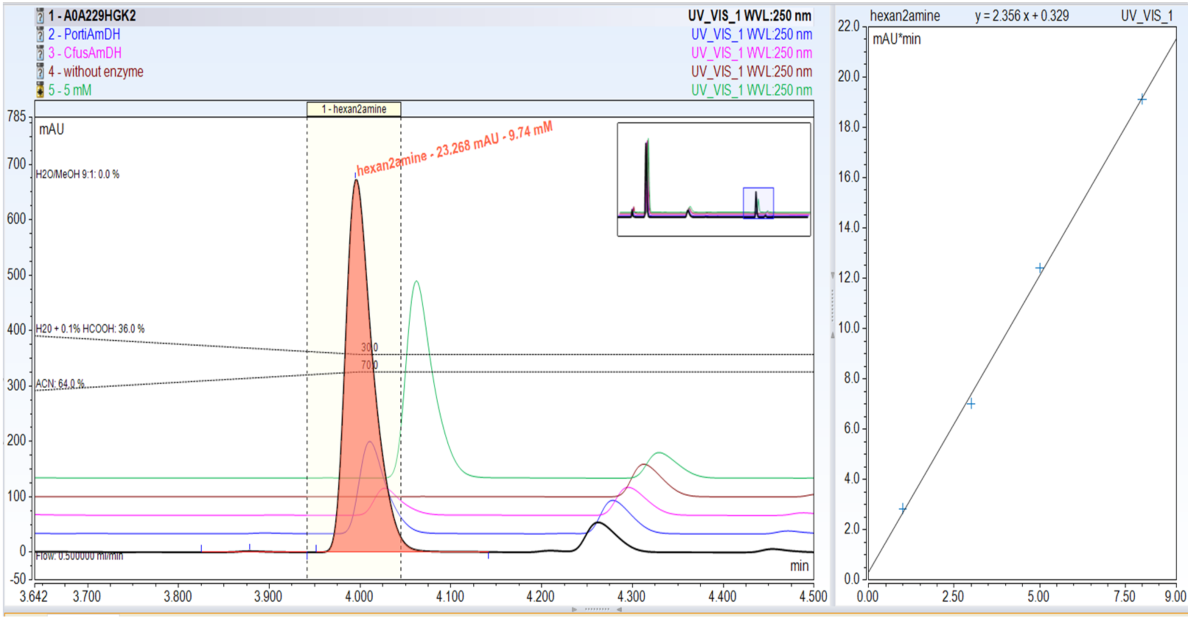

Supplementary Figure 14. UV-chromatograms (250 nm) of benzoyl-derivatized enzymatic reaction mixtures with purified MGYP001209562846, A0A229HGK2, *Porti*AmDH, *Cfus*AmDH, blank reaction mixture without enzyme and one calibration point of amine for each corresponding tested ketones: (A) hexan-3-one (**5a**), (B) heptan-3-one (**13a**), (C) hexan-2-one (**12a**). The amount of each amine was deduced from the calibration curve provided in the Figure.

A

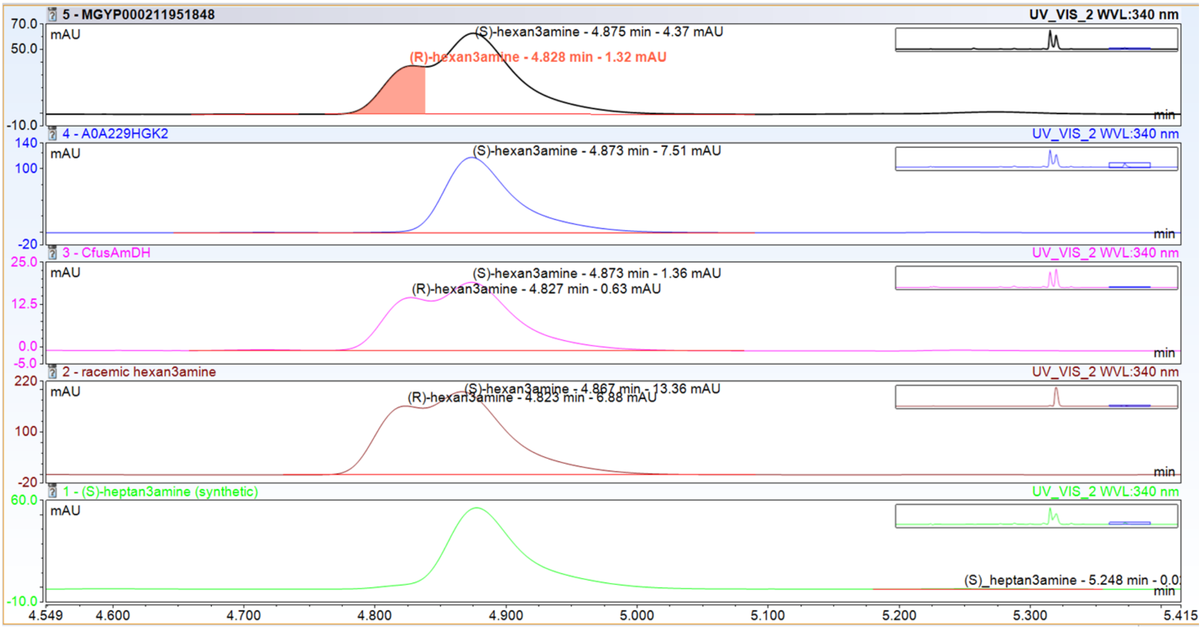

B

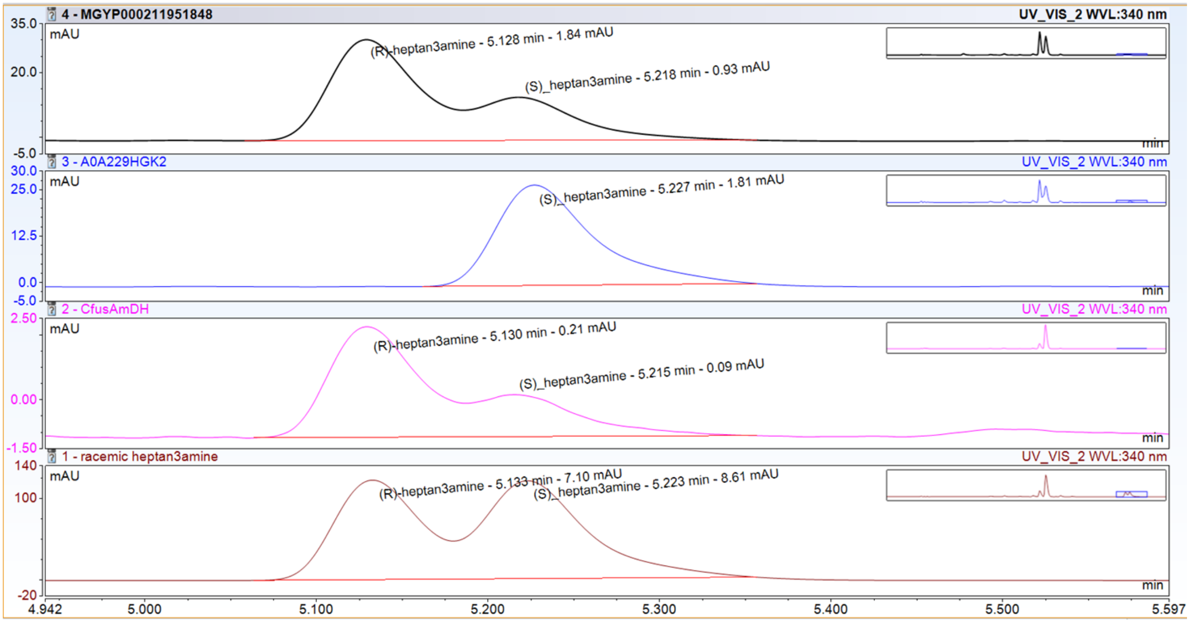

C

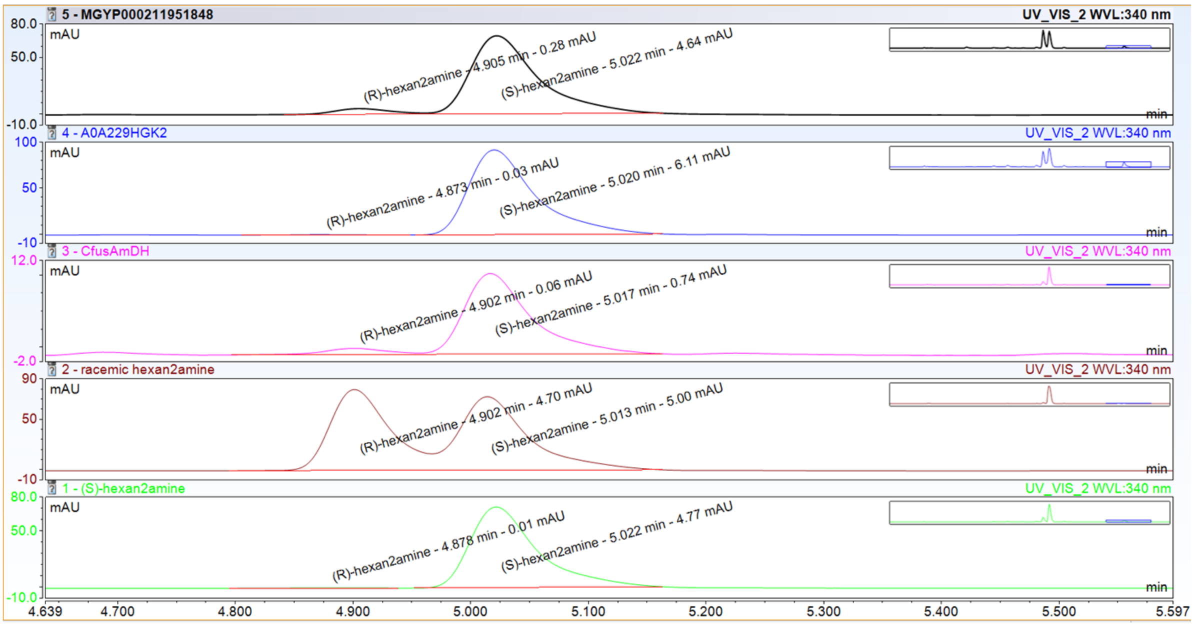

Supplementary Figure 15. UV-chromatograms (340 nm) of FDAA-derivatized enzymatic reaction mixtures with purified MGYP000211951848, A0A229HGK2, *Cfus*AmDH, racemic amine **5b**, **12b**-**13b** and commercial or synthetical enantiomerically enriched amine **5b**, **12b**-**13b** for reaction with (A) hexan-3-one (**5a**), (B) heptan-3-one (**13a**) and (C) hexan-2-one (**12a**).

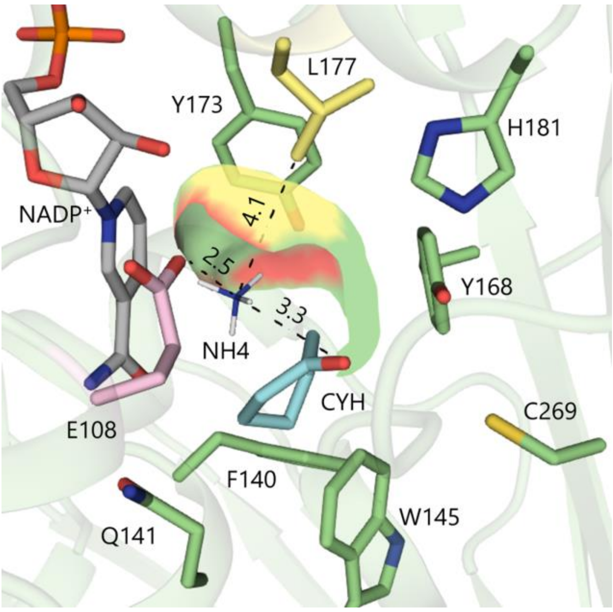

Supplementary Figure 16. *Cfus*AmDH active site with docking of cyclohexanone (**2a**) (CYH) and ammonia (**b**) (NH4). The surface of the upper part of the catalytic pocket is represented. L177 is highlighted in yellow and some of the P1-P21 positions are represented in green. NADP$^+$ and **2a** are colored in grey and blue, respectively. Distances are given in Ångströms (Å).

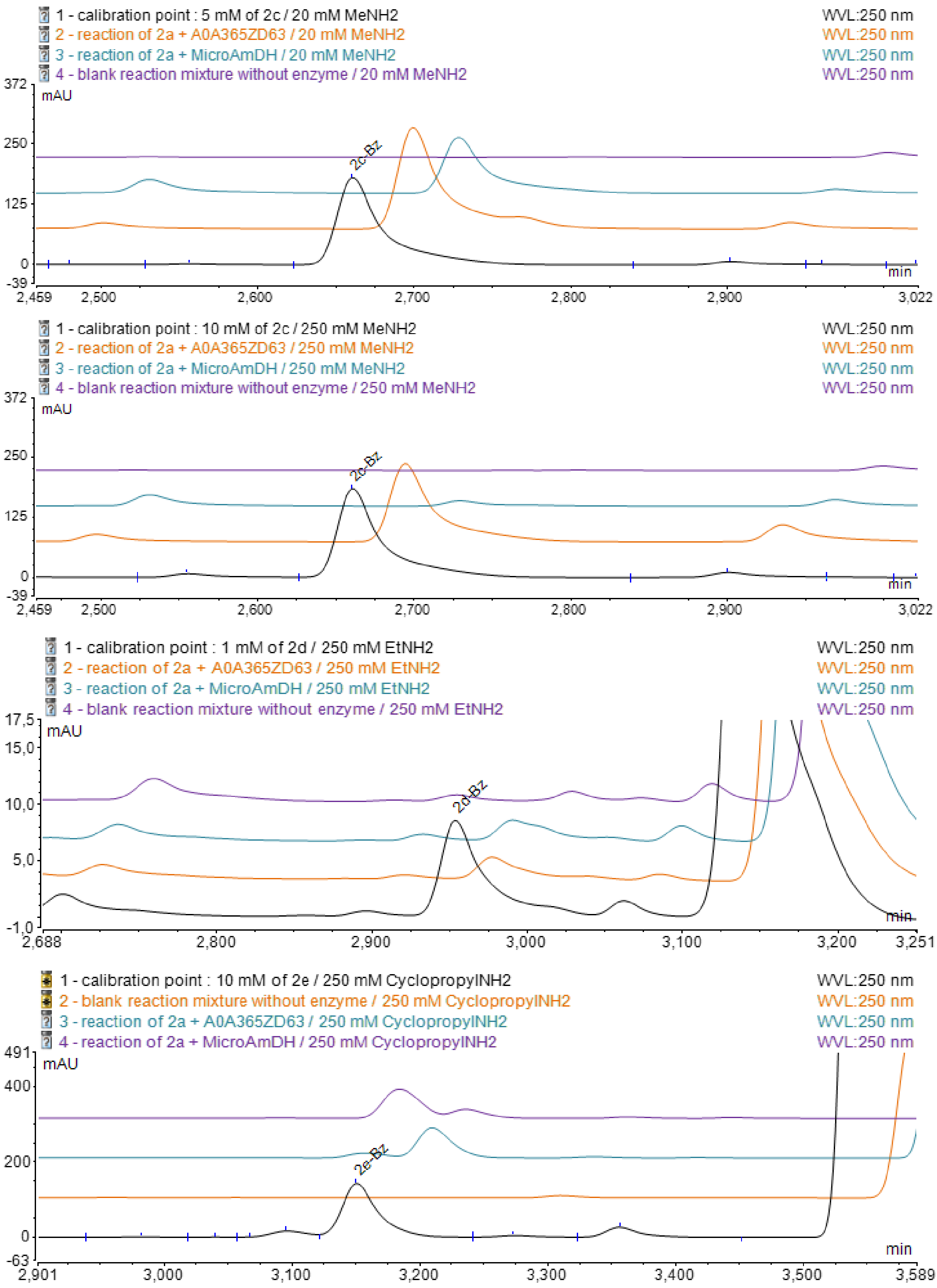

Supplementary Figure 17. UV-chromatograms (250 nm) of benzoyl-derivatized enzymatic reaction mixtures with purified A0A365ZD63, *Micro*AmDH, blank reaction mixture without enzyme and one calibration point of *N*-alkylamine for reaction of **2a** with each corresponding tested amine: methylamine (**c**) at 20 mM, methylamine (**c**) at 250 mM, ethylamine (**d**) at 250 mM and cyclopropylamine (**e**) at 250 mM. The amount of each amine was deduced from calibration curves obtained with standard amines.

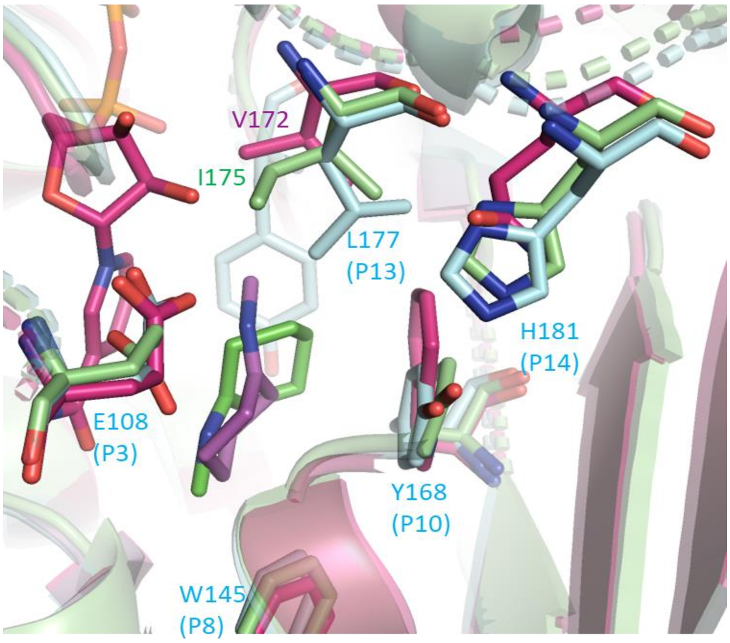

Supplementary Figure 18. PyMOL visualization of the active site of *Cfus*AmDH (light blue), A0A365ZD63 (pink) and IGC-32 (green) with corresponding docked *N*-methylcyclohexyliminium (green in IGC-32 and purple in A0A365ZD63). Only positions P3, P8, P10, P13 and P14 are represented for clarity.

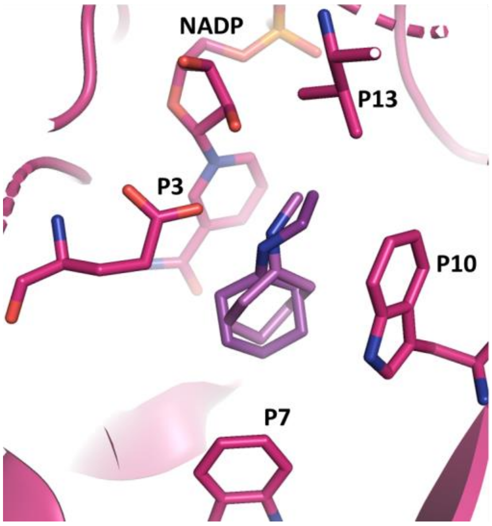

Supplementary Figure 19. PyMOL visualization of the active site of A0A365ZD63 (pink) with corresponding docked *N*-methylcyclohexyliminium and *N*-ethylcyclohexyliminium (purple). Only positions P3, P7, P10 and P13 are represented for clarity.

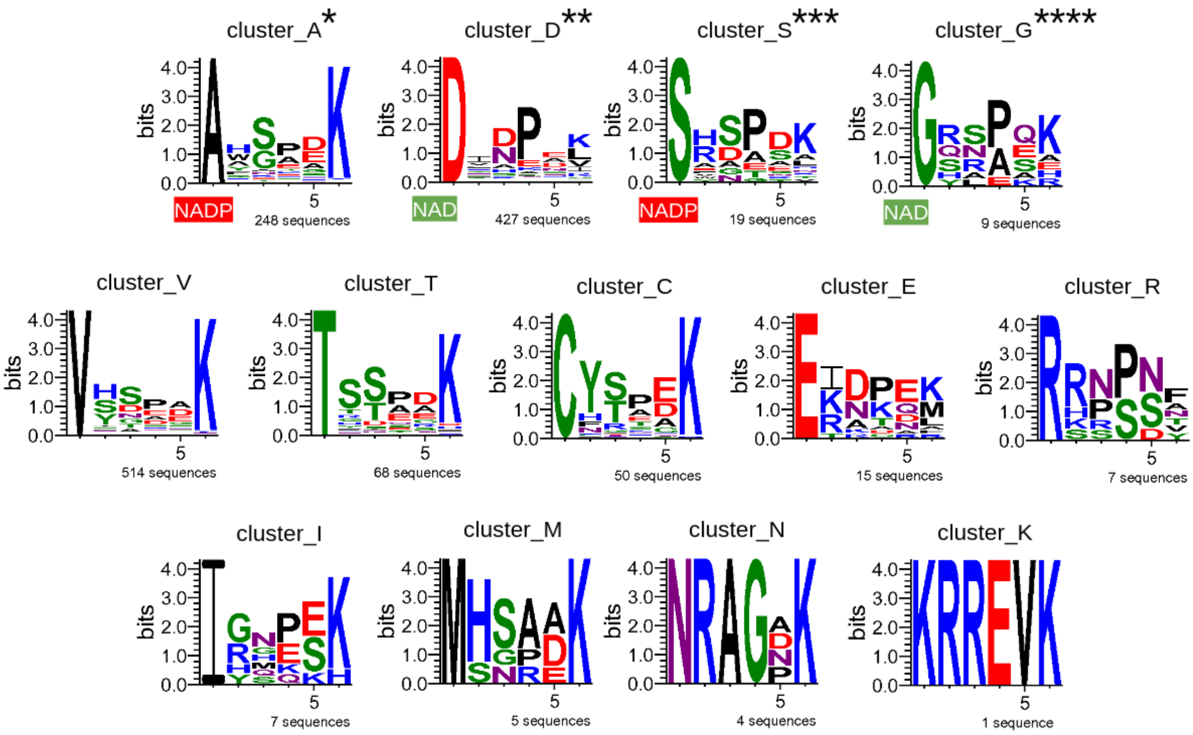

Supplementary Figure 20. Sequence-based clustering of NAD(P)-binding patterns under positions D36-R41 of *Cfus*AmDH. Cofactor preference is indicated for clusters A, D, S and G for which we have experimental data: (*) *Myco*AmDH, *Mvac*AmDH, *Msme*AmDH, AmDH9; (**) *Acol*AmDH, *Chat*AmDH, *Sgor*AmDH, *Apau*AmDH, *Cfus*AmDH, *Tther*AmDH, AmDH2, AmDH4, AmDH8, IGCAmDH1, IGCAmDH5, MATOUAmDH1, MATOUAmDH2; (***) *Micro*AmDH; (****) AmDH5, AmDH7.

Supplementary Methods

Screening of NAD(P)-dependent enzymes among biodiversity

We used the 301 SCOP Superfamily HMM signatures of the NAD(P)-binding Rossmann-fold domain (SCOP ID: SSF51735) to build the library of NAD(P)-dependent enzymes. This signature is integrated into the InterPro entry IPR036291 (“NAD(P)-binding domain superfamily”), also used to annotate UniProtKB sequences.

Then, both SwissProt (release 2020_02) and TrEMBL (release 2020_03) databases were screened using these HMM signatures and a cut-off score was determined by classifying enzyme hits as follows:

- True Positive (TP): score > cutoff AND with IPR036291 annotation
- False Positive (FP): score > cutoff AND without IPR036291 annotation
- True Negative (TN): score < cutoff AND without IPR036291 annotation
- False Negative (FN): score < cutoff AND with IPR036291 annotation

Supplementary Table 11 presents the total number of hits retrieved from SwissProt and TrEMBL and their respective TP/FP/TN/FN classification under different cutoffs.

The cutoff score was selected as a balance between sensitivity, computed as TP / (TP + FN), and specificity, computed as TN / (TN + FP): based on the results, the cutoff was set to 50.

One may have set the cutoff score at 100 but, here, we aimed to reduce the initial set of NAD(P)-dependent enzymes in a way suitable for the Superfamily assignment script in order to minimize the computation time.

Screening of Amine Dehydrogenases (AmDHs) among biodiversity

Starting from the former AmDH set[4], new AmDHs were collected among metagenomic protein databases using a three-step protocol. This required the definition of selection criteria, to keep only relevant AmDH sequences, which was performed by screening the TrEMBL resource (release 2020_03).

a) Active site signature

A C-terminus (here, the active site) signature was designed for the whole family (all_ASMC_no_nad) to screen metagenomic databases. This was conducted by removing the NAD(P)-binding domain pattern of the AmDH4 sequence (UniProt ID: A9BHL2) from the corresponding multiple sequence alignment.

To analyze the screening results, we identified the former AmDH sequences among the potential new ones. A cut-off score was defined as the value of the minimum score found for an AmDH, here a score of 77.6 (max: 240.7). However, only 9 out of 1,815 sequences had a score lower than 100.

Based on these results and to avoid bias in the active site description due to potential false positives, we chose a cutoff value of 100, instead of 50, as it resulted in the recovery of 1,806 out of 1,815 ASMC sequences, namely 99.5% of the former AmDH set (the 9 missing sequences being those with a score lower than 100, as mentioned before).

b) Sequence length limitations

To anticipate the protein expression and production steps and given the size of the NAD(P)-binding domain (here, ~150 amino acids), we only kept AmDH sequences between 250 and 500 amino acids in length.

c) NAD(P)-binding domain

A N-terminus (here, the NAD(P)-binding domain) signature was designed for the whole family (all_ASMC_nad_dom) to screen the set of hit sequences with a score greater than 100 from the previous C-terminus screening. This was conducted by removing the active site pattern of the AmDH4 sequence (UniProt ID: A9BHL2) from the corresponding multiple sequence alignment.

As for the active site signature, a cutoff score was defined as the minimum score to gather all AmDHs, here a score of 53.2 (max: 196.0). This time only 13 out of 1,815 sequences had a score lower than 100.

Based on this result and given the global high diversity of NAD(P)-binding domains (i.e. 301 HMM profiles in SCOP Superfamily), we decided to not round up the cutoff to 100 but to round it down to 50.

Supplementary Discussion

Cofactor specificity of nat-AmDHs

Cluster_D and cluster_E, including 1067 and 20 sequences respectively, should correspond to NADH-enzymes as position R1 is occupied by a negatively charged residue (Asp, Glu). Indeed, cluster_D gathers *Acol*AmDH, *Chat*AmDH, *Sgor*AmDH, *Apau*AmDH, IGCAmDH1, IGCAmDH5, but also the 2,4-DAPDHs AmDH2, AmDH4, AmDH8 and *Tther*AmDH, all experimentally validated to display better activity with NADH over NADPH cofactor[3-5]. In the AmDH4 structure (PDB ID: 6g1m, chain B), Asp33 (R1) strongly interacts with both 2'OH and 3'OH of the ribose while Phe34 (R2) makes a pi-staggered interaction (parallel displaced) stabilizing the adenine moiety and Tyr38 (R6) being in pi-teeing interaction (perpendicular T-shaped) with Phe9. Otherwise, the steric hindrance induced by the presence of Arg35 (R3) could disrupt the fixation of NADPH. Interestingly, *Cfus*AmDH is also present in cluster_D while accepting both NADH and NADPH cofactors. Based on its X-ray structure (PDB ID: 6iau, chain B), residues His37 (R2) and Asn38 (R3) interact with the 2'P group while Asp36 (R1) and Arg41 (R6) interact with the 3'OH. Regarding the NADPH-nat-AmDHs, we explored the active site of *Msme*AmDH (PDB ID: 6iaq, chain C) and found that 3'OH of the ribose has no interaction (Ala34 in R1) whereas 2'P is surrounded by three residues in R2, R3 and R6 positions (Arg35, Ser36 and Lys39, respectively). Serine, here in R3 position, is known for stabilizing the NADPH cofactor through its interaction with the phosphate group and its short side chain prevents interaction with the OH groups of NADH-adenine moiety; this is why replacement with Asp or Arg allows NADPH to be used instead of NADH[6].

Supplementary References

1. Madeira, F. *et al.* Search and sequence analysis tools services from EMBL-EBI in 2022. *Nucleic Acids Res.* **50**, W276–W279 (2022).
2. Stam, M. *et al.* NetSyn: genomic context exploration of protein families. *bioRxiv* (2023) doi:10.1101/2023.02.15.528638.
3. Mayol, O. *et al.* Asymmetric reductive amination by a wild-type amine dehydrogenase from the thermophilic bacteria Petrotoga mobilis. *Catal. Sci. Technol.* **6**, 7421–7428 (2016).
4. Mayol, O. *et al.* A family of native amine dehydrogenases for the asymmetric reductive amination of ketones. *Nat. Catal.* **2**, 324–333 (2019).
5. Caparco, A. A. *et al.* Metagenomic mining for Amine dehydrogenase discovery. *Adv. Synth. Catal.* **362**, 2427–2436 (2020).
6. Chánique, A. M. & Parra, L. P. Protein Engineering for Nicotinamide Coenzyme Specificity in Oxidoreductases: Attempts and Challenges. Front. Microbiol. 9, 194 (2018).
