# Supplementary material for: A refined picture of the native amine dehydrogenase family revealed by extensive biodiversity screening
Source: Nat Commun. 2024 Jun 10;15:4933. doi: 10.1038/s41467-024-49009-2 (PMC11164908; doi:10.1038/s41467-024-49009-2)
Supplement: Supplementary file 3 — Description of additional supplementary files [file 41467_2024_49009_MOESM3_ESM.pdf]

<u>Description of Additional Supplementary Files</u>

Supplementary Data 1
Description: List of 1,090 amines used for the virtual screening in the G1-enzyme from *Vulcanisaeta distributa* (UniProt ID: E1QRK4).
PubChem ID and SMILES are provided.

Supplementary Data 2
Description: Activity screening results of selected G1 members.
a: "+" indicates a clear band on SDS-gel using Nu-PAGE system (Invitrogen).
b: total protein concentrations determined by the Bradford method with bovine serum albumin as the standard
nd: not detected.
Tested substrates with positive hits (2-amino-4-oxopentanoic acid. HCl (**1a**) and cyclohexanone (**2a**)) are detailed. The results with the other tested substrates (4-oxocyclohexane-1,1-dicarboxylic acid; piperidine dicarboxylate; diaminopimelate; 2,6-diamino-5-hydroxyhexanoic acid. HCl; *N*-(2-aminoethyl)glycine, see Supplementary Table 4) for which no significant positive responses were detected, were not mentioned in this Figure.
Screening results are indicated as the slope in mU min^{-1} obtained between 0-500 s after protein addition in case of the NAD(P)H spectrophotometric assay, or as the qualitative amount (mM) of corresponding amine formed deduced from calibration curves following UHPLC-UV analysis. 2-amino-4-oxopentanoic acid.HCl (**1a**) and cyclohexanone (**2a**) were tested in the reductive way, the others in the oxidative way (see details in Methods). Cells highlighted in green correspond to positive hits (darker green for higher activity). "PET Coli" corresponds to crude cell lysate without any overexpressed protein. Reference enzymes correspond to enzymes already described in previous published work: AmDH4, D2RES1 and B9KZJ1[3,4].

Supplementary Data 3
Description: Activity screening results of selected G2 members.
a: "+" indicates a clear band on SDS-gel using Nu-PAGE system (Invitrogen).
b: total protein concentrations determined by the Bradford method with bovine serum albumin as the standard.
c: non representative slope due to high consumption of NAD(P)H in the first seconds before triggering OD recording (high hits).
nd: not detected.
Substrates (2-amino-4-oxopentanoic acid.HCl (**1a**); cyclohexanone (**2a**)) were tested in the reductive way. Screening results are indicated as the slope in mU min^{-1} obtained between 0-500 s after protein addition in case of the NAD(P)H spectrophotometric assay, or as the qualitative amount (mM) of corresponding amine formed deduced from calibration curves following UHPLC-UV analysis (see details in Methods). Cells highlighted in green correspond to positive hits (darker green for higher activity). "PET Coli" corresponds to crude cell lysate without any overexpressed protein. Reference enzymes correspond to enzymes already described in previous published work: AmDH4, D2RES1 and *Msme*AmDH[3-4].

Supplementary Data 4

Description: Activity screening results of selected G3-G5 members.

a: "+" indicates a clear band on SDS-gel using Nu-PAGE system (Invitrogen).

b: total protein concentrations determined by the Bradford method with bovine serum albumin as the standard.

nd: not detected; nt: not tested; t: traces of detected product; TPC: total protein concentration; *: activity not confirmed on purified enzyme.

Substrates were tested in the reductive way. Screening results are indicated as the slope in mU min^{-1} obtained between 0-500 s after protein addition in case of the NAD(P)H spectrophotometric assay, or as the qualitative amount (mM) of corresponding amine formed deduced from calibration curves following UHPLC-UV analysis (see details in Methods). Cells highlighted in green correspond to positive hits (darker green for higher activity). "PET Coli" corresponds to crude cell lysate without any overexpressed protein. Reference enzymes correspond to enzymes already described in previous published work: AmDH4, D2RES1, *Msme*AmDH and *Cfus*AmDH and *Porti*AmDH[3-5].

Supplementary Data 5

Description: List of the 122 enzymes selected from the ref-AmDH family and their P1-P21 positions. P1-P21 positions are indicated in separated tabs according to the reference structure used: AmDH4, for G1 and G2 groups, or *Cfus*AmDH and *Msme*AmDH, for G1, G3, G4 and G5 groups. "–" means that the ASMC pipeline did not find any amino acid superimposed on the reference in the corresponding models.

(a): In case of close homologs (seq id > 95%) present in the UniProtKB database, the UniProt homolog has been produced instead of the original sequence. The protein ID has been subsequently formatted as follows: original sequence/produced sequence.

Supplementary Data 6

Description: Sequence identity matrix of the 72 *in vitro* validated AmDHs together with previously reported nat-AmDHs (red-labeled).

Reference AmDHs are color-labeled in red and corresponding ASMC groups are indicated in the second column.

Identity percentages are colored under the following color scheme: [0;50[: grey, [50;70[: green, [70;90[: yellow, [90;100[: orange, [100]: red.

Supplementary Data 7

Description: List of purified AmDHs tested for specific features. Active site key positions (P1-P21) are indicated.

LP: larger pocket; 3C: carbonyl at position 3 on the alkyl chain. Cells colored in green and red referred to detected activity and undetected activity respectively. "objectives" refers to the goal of the selected sequences, either activity regarding the carbonyl substrate scope (« LP » and « 3C »), or the amine substrate scope (« amine »). The selection has been carried out using the enlarged set of ref-AmDHs (9,886 models).

*: No residue at the position P7 and P8. This enzyme has been selected due to the absence of two helix turns at this spatial position resulting in a larger pocket.

Supplementary Data 8

Description: Docking results of (3*R*)- and (3*S*)-heptan-3-amine (**13b**) in *Cfus*AmDH, A0A229HGK2, MGYP000211951848.

Supplementary Data 9

Description: Sequence identity matrix of the 17 *in vitro* validated AmDHs with specific features together with previously reported nat-AmDHs (red-labeled).

Protein IDs are color-labeled in blue for corresponding enzymes active towards bulky substrates, in green for corresponding enzymes active towards 3C-ketones and in pink for corresponding enzymes active with alkylamines.Identity percentages are colored under the following color scheme: [0;50[: grey, [50;70[: green, [70;90[: yellow, [90;100[: orange, [100]: red.

Supplementary Data 10

Description: List of 5' and 3' primers used to produce the enzymes presented in this study.
