# Supplementary material for: A refined picture of the native amine dehydrogenase family revealed by extensive biodiversity screening
Source: Nat Commun. 2024 Jun 10;15:4933. doi: 10.1038/s41467-024-49009-2 (PMC11164908; doi:10.1038/s41467-024-49009-2)
Supplement: Supplementary file 11 — Supplementary Data 8 [file 41467_2024_49009_MOESM11_ESM.docx]

1. Docking results of (3*R*)- and (3*S*)-heptan-3-amine (**13b**) in *Cfus*AmDH, A0A229HGK2, MGYP000211951848.

|  | *S*-heptan-3-amine ((*S*)-**13b**) | | *R*-heptan-3-amine ((*R*)-**13b**) | |
| --- | --- | --- | --- | --- |
| **# conformations** | **Energy of binding**  **(kcal mol^-1^)** | **Correct orientation?** | **Energy of binding**  **(kcal mol^-1^)** | **Correct orientation?** |
| *CfusAmDH* | | | | |
| 1 | -6.29 | N | -6.36 | Y |
| 2 | -6.28 | N | -6.35 | N |
| 3 | -6.27 | N | -6.26 | Y |
| 4 | -6.27 | Y | -6.26 | N |
| 5 | -6.25 | N | -6.25 | N |
| 6 | -6.24 | Y | -6.24 | N |
| 7 | -6.23 | N | -6.23 | N |
| 8 | -6.23 | N | -6.22 | N |
| 9 | -6.23 | N | -6.22 | Y |
| 10 | -6.21 | N | -6.22 | N |
| 11 | -6.21 | N | -6.21 | N |
| 12 | -6.20 | N | -6.18 | N |
| 13 | -6.19 | N | -6.17 | N |
| 14 | -6.19 | N | -6.17 | N |
| 15 | -6.17 | N | -6.12 | N |
| 16 | -6.07 | N | -6.11 | N |
| 17 | -6.07 | N | -6.1 | N |
| 18 | -6.06 | Y | -6.04 | N |
| 19 | -5.88 | N | -5.99 | N |
| 20 | -5.86 | Y | -5.94 | N |
| **Average for correct conformations:** | **-6.11** | **4** | **-6.28** | **3** |
| *A0A229HGK2* | | | | |
| 1 | -6.77 | Y | -6.72 | N |
| 2 | -6.76 | N | -6.72 | N |
| 3 | -6.73 | Y | -6.7 | N |
| 4 | -6.72 | N | -6.68 | N |
| 5 | -6.72 | Y | -6.66 | N |
| 6 | -6.67 | Y | -6.66 | Y |
| 7 | -6.67 | N | -6.62 | N |
| 8 | -6.67 | Y | -6.58 | N |
| 9 | -6.66 | Y | -6.56 | Y |
| 10 | -6.66 | Y | -6.55 | N |
| 11 | -6.64 | Y | -6.54 | Y |
| 12 | -6.63 | Y | -6.54 | N |
| 13 | -6.62 | Y | -6.53 | N |
| 14 | -6.61 | Y | -6.5 | N |
| 15 | -6.56 | Y | -6.49 | N |
| 16 | -6.54 | Y | -6.46 | N |
| 17 | -6.44 | Y | -6.46 | N |
| 18 | -6.44 | Y | -6.45 | N |
| 19 | -6.39 | Y | -6.43 | N |
| 20 | -6.38 | Y | -6.38 | N |
| **Average for correct conformations:** | **-6.60** | **17** | **-6.59** | **3** |
|  | | | | |

1. Docking results of (3*R*)- and (3*S*)-heptan-3-amine (**13b**) in *Cfus*AmDH, A0A229HGK2, MGYP000211951848. (continued)

|  | *S*-heptan-3-amine ((*S*)-**13b**) | | *R*-heptan-3-amine ((*R*)-**13b**) | |
| --- | --- | --- | --- | --- |
| **# conformations** | **Energy of binding**  **(kcal mol^-1^)** | **Correct orientation?** | **Energy of binding**  **(kcal mol^-1^)** | **Correct orientation?** |
| *MGYP000211951848* | | | | |
| 1 | -7.25 | Y | -7.22 | Y |
| 2 | -7.16 | N | -7.2 | Y |
| 3 | -7.08 | Y | -7.19 | N |
| 4 | -7.04 | Y | -7.18 | N |
| 5 | -6.94 | Y | -7.18 | N |
| 6 | -6.93 | N | -7.17 | Y |
| 7 | -6.88 | Y | -7.17 | N |
| 8 | -6.86 | Y | -7.12 | N |
| 9 | -6.85 | Y | -7.11 | Y |
| 10 | -6.84 | Y | -7.08 | N |
| 11 | -6.83 | Y | -6.99 | N |
| 12 | -6.82 | N | -6.88 | N |
| 13 | -6.8 | N | -6.87 | N |
| 14 | -6.8 | Y | -6.86 | Y |
| 15 | -6.79 | N | -6.85 | Y |
| 16 | -6.79 | Y | -6.8 | Y |
| 17 | -6.77 | N | -6.77 | N |
| 18 | -6.76 | Y | -6.77 | Y |
| 19 | -6.7 | Y | -6.68 | Y |
| 20 | -6.69 | N | -6.59 | Y |
| **Average for correct conformations:** | **-6.89** | **13** | **-6.93** | **10*** |

“Correct” conformations (Y = yes; N = no) correspond to conformations within the 20 calculated ones where the delivered hydrogen of the docked amine (C**H**-NH_2_) is oriented face to C4 nicotinamide ring with distance < 4.5 Å.  Energies of binding are expressed in kcal mol^-1^. *: NH_2_ is quite far from C4 nicotinamide. For “correct” conformations, the average of energies of binding are provided. Their numbers were higher for enzymes giving higher analytical yields (23/40 and 20/40 for MGYP000211951848 and A0A229HGK2 respectively) *versus* less active ones (7/40 for *Cfus*AmDH). The ratio of (*S*)-correct conformations *vs* correct (*R*)-ones was much higher for the highly *S*-selective A0A229HGK2 compared to MGYP000211951848 (17/3 compared to 13/10). In *Cfus*AmDH, the conformations appear more constrained by a smaller available space and P3/P17 positioning, which resulted in higher energies (-6.11 kcal mol^-1^ compared to -6.60 and -6.89 kcal mol^-1^ for A0A229HGK2 and MGYP000211951848, respectively) and therefore much lower analytical yields.

1. Examples of correct and incorrect conformations (PyMOL visualization)

| Example in *Cfus*AmDH of correct (pink) and incorrect (green) conformations | 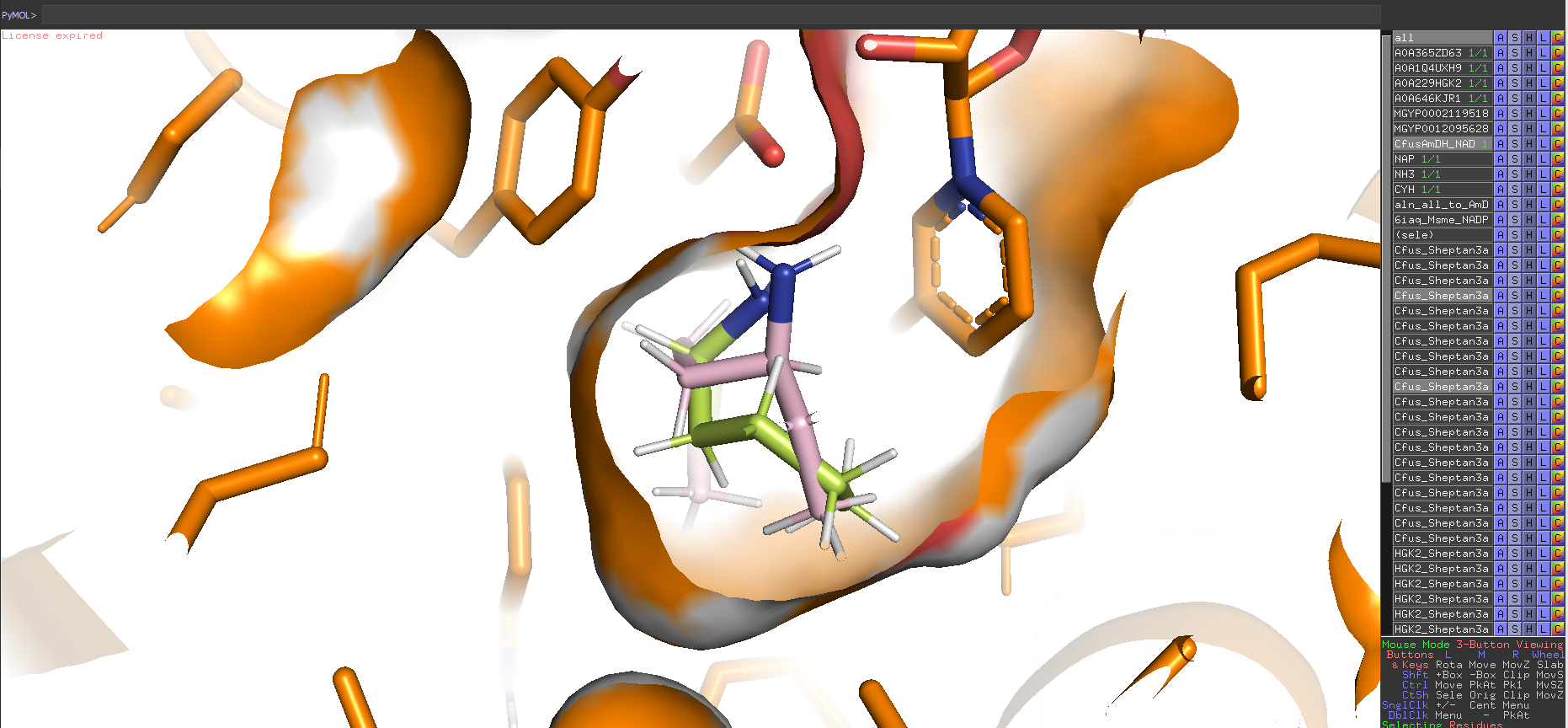 |
| --- | --- |
| Example in A0A229HGK2 of correct (blue) and incorrect (green) conformations | 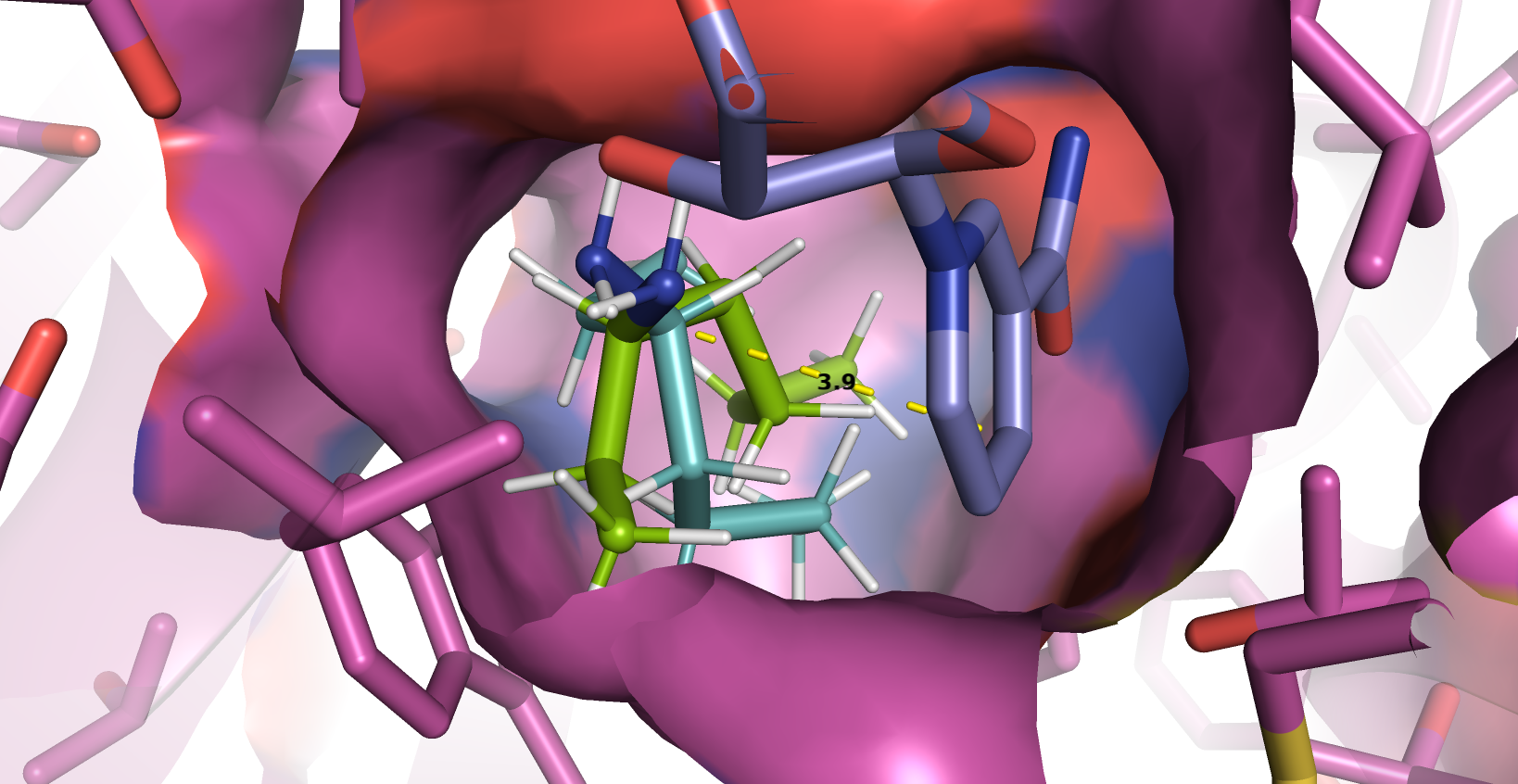 |
