# Supplementary material for: A refined picture of the native amine dehydrogenase family revealed by extensive biodiversity screening
Source: Nat Commun. 2024 Jun 10;15:4933. doi: 10.1038/s41467-024-49009-2 (PMC11164908; doi:10.1038/s41467-024-49009-2)
Supplement: Supplementary file 14 — Reporting Summary [file 41467_2024_49009_MOESM14_ESM.pdf]

nature portfolio

Corresponding author(s): Carine Vergne-Vaxelaire
David Vallenet

Last updated by author(s): Apr 5, 2024

Reporting Summary

Nature Portfolio wishes to improve the reproducibility of the work that we publish. This form provides structure for consistency and transparency in reporting. For further information on Nature Portfolio policies, see our Editorial Policies and the Editorial Policy Checklist.

Statistics

For all statistical analyses, confirm that the following items are present in the figure legend, table legend, main text, or Methods section.

n/a	Confirmed	
☐	☒	The exact sample size (*n*) for each experimental group/condition, given as a discrete number and unit of measurement
☒	☐	A statement on whether measurements were taken from distinct samples or whether the same sample was measured repeatedly
☒	☐	The statistical test(s) used AND whether they are one- or two-sided<br>*Only common tests should be described solely by name; describe more complex techniques in the Methods section.*
☒	☐	A description of all covariates tested
☒	☐	A description of any assumptions or corrections, such as tests of normality and adjustment for multiple comparisons
☒	☐	A full description of the statistical parameters including central tendency (e.g. means) or other basic estimates (e.g. regression coefficient) AND variation (e.g. standard deviation) or associated estimates of uncertainty (e.g. confidence intervals)
☒	☐	For null hypothesis testing, the test statistic (e.g. *F*, *t*, *r*) with confidence intervals, effect sizes, degrees of freedom and *P* value noted<br>*Give P values as exact values whenever suitable.*
☒	☐	For Bayesian analysis, information on the choice of priors and Markov chain Monte Carlo settings
☒	☐	For hierarchical and complex designs, identification of the appropriate level for tests and full reporting of outcomes
☒	☐	Estimates of effect sizes (e.g. Cohen's *d*, Pearson's *r*), indicating how they were calculated

Our web collection on statistics for biologists contains articles on many of the points above.

Software and code

Policy information about availability of computer code

Data collection	Construction of HMM profiles and protein sequence searches in (meta-)genomic databases were carried out using the HMMER (v3.3) and HHpred (v3.0.3) suites.
Data analysis	Protein sequences were annotated using the SCOP Superfamily script [https://supfam.mrc-lmb.cam.ac.uk/SUPERFAMILY/howto_use_models.html], clustered using MMseqs2 (version 12.git113e321) and CD-HIT (v4.6), aligned and trimmed with MAFFT (v7.464) and TrimAl (v1.2), respectively. Sequence logos were designed using WebLogo (v3.0). HMM profiles were compared using HHblits (v3.0.3). Phylogenetic trees were built with IQ-TREE (v1.6.12) and visualized within the iTOL online software [https://doi.org/10.1093/nar/gkab301]. Active site comparison was conducted using ASMC [https://github.com/labgem/asmc_2016] and genomic context using NetSyn [https://github.com/labgem/netsyn]. Energy minimization, virtual screening and docking experiments were carried out using YASARA Structure (v22.9.24). Docking experiments were also done with AutoDockTool. 3D-models were visualized using PyMOL Molecular Graphics System, Version 2.5 Schrödinger, LLC (version 2.5-master-d24468af).

For manuscripts utilizing custom algorithms or software that are central to the research but not yet described in published literature, software must be made available to editors and reviewers. We strongly encourage code deposition in a community repository (e.g. GitHub). See the Nature Portfolio guidelines for submitting code & software for further information.

Data

Policy information about availability of data

All manuscripts must include a data availability statement. This statement should provide the following information, where applicable:

- Accession codes, unique identifiers, or web links for publicly available datasets
- A description of any restrictions on data availability
- For clinical datasets or third party data, please ensure that the statement adheres to our policy

Data generated in this study can be accessed through the Zenodo repository [https://doi.org/10.5281/zenodo.7889419]. It contains libraries of NAD(P)-dependent enzyme sequences and ref-AmDH sequences, HMM libraries of NAD(P)-dependent protein subfamilies and nat-AmDHs, ref-AmDH homology models, as well as sequences of representative ref-AmDHs tested and of heterologously expressed nat-AmDHs with specific feature. PDB accessions were obtained from RCSB PDB [https://www.rcsb.org/] and include 6G1M [https://www.rcsb.org/structure/6G1M], 6IAU [https://www.rcsb.org/structure/6IAU], 6IAQ [https://www.rcsb.org/structure/6IAQ] and 7ZBO [https://www.rcsb.org/structure/7ZBO]. Protein sequences were extracted from the genomic and metagenomic databases listed in the Supplementary Table 1. All other data supporting the findings of this study are available within the paper and its Supplementary Information and Data. Source data are provided with this paper.

Research involving human participants, their data, or biological material

Policy information about studies with human participants or human data. See also policy information about sex, gender (identity/presentation), and sexual orientation and race, ethnicity and racism.

Reporting on sex and gender	only genomic data and enzymes are considered in this work,so sex and gender are not parameters to be considered
Reporting on race, ethnicity, or other socially relevant groupings	only genomic data and enzymes are considered in this work,so race, ethnicity or other socially groupings are not relevant to consider
Population characteristics	only genomic data and enzymes are considered in this work,so population characteristics were not considered
Recruitment	only genomic data and enzymes are considered in this work,so recruitment was not considered
Ethics oversight	only genomic data and enzymes are considered in this work,so ethics oversight was not relevant to consider

Note that full information on the approval of the study protocol must also be provided in the manuscript.

Field-specific reporting

Please select the one below that is the best fit for your research. If you are not sure, read the appropriate sections before making your selection.

☒ Life sciences ☐ Behavioural & social sciences ☐ Ecological, evolutionary & environmental sciences

For a reference copy of the document with all sections, see nature.com/documents/nr-reporting-summary-flat.pdf

Life sciences study design

All studies must disclose on these points even when the disclosure is negative.

Sample size	No sample size calculation was performed. Given the size of the nat-AmDH family (17,959 sequences), representative enzymes were selected with the support of in silico analysis (sequence identity, comparison of active sites, phylogeny) to cover each family subgroup and reduce the number of experiments to be performed.
Data exclusions	In term of molecular modeling, all the obtained docking poses were analyzed based on scoring values of Autodock's scoring function as described in Supplementary Information. The best docking pose (i.e. rank first according to Autodock scoring function) were chosen. We discarded some sequences based on various criteria all detailed in Methods in the main text (old sequences, those containing less than 250 or more than 500 amino acids, redundancy, family hits if probability scores were greater than or equal to 95%, sequences without glutamate in P3 for the selection of representative enzymes...). No other data excluded.
Replication	Enzyme activity screening was not repeated, except for selected candidates with potential activity for the targeted substrates, for which additional activity assays (n=2) were performed on purified enzymes, as described in the Supplementary Information and Methods. Regarding the in silico experiments, software parameters are described in the Methods section and Supplementary Information to help reproduce the corresponding results.
Randomization	No randomization was applied to the data. The enzymes collected in this study were assigned to an experimental group on the basis of in silico (sequence identity, active site comparison) and experimental (activity screening) analyses, and by comparing them with data already collected for each AmDH group by Mayol et al. [https://doi.org/10.1038/s41929-019-0249-z].<br>Only recombinant proteins and E. coli cells were involved in this study (no animal or human participant).

Blinding

Data collection based on genomic criteria was blind, as we searched for any NAD(P)-dependent enzyme, regardless of the enzymatic reaction performed. However, the updating of the AmDH family, the selection of specific enzymes within this family and the selection of substrates were not carried out blindly, as the AmDH features were necessary to set up the in silico and in vitro experiments described in the "Methods" section.

Reporting for specific materials, systems and methods

We require information from authors about some types of materials, experimental systems and methods used in many studies. Here, indicate whether each material, system or method listed is relevant to your study. If you are not sure if a list item applies to your research, read the appropriate section before selecting a response.

Materials & experimental systems

n/a	Involved in the study
☒	☐ Antibodies
☒	☐ Eukaryotic cell lines
☒	☐ Palaeontology and archaeology
☒	☐ Animals and other organisms
☒	☐ Clinical data
☒	☐ Dual use research of concern
☒	☐ Plants

Methods

n/a	Involved in the study
☒	☐ ChIP-seq
☒	☐ Flow cytometry
☒	☐ MRI-based neuroimaging

Plants

Seed stocks

the study did not involve seed stocks

Novel plant genotypes

the study did not involve novel plant genotypes

Authentication

Describe any authentication procedures for each seed stock used or novel genotype generated. Describe any experiments used to assess the effect of a mutation and, where applicable, how potential secondary effects (e.g. second site T-DNA insertions, mosiacism, off-target gene editing) were examined.
