# Supplementary figures and images for: A refined picture of the native amine dehydrogenase family revealed by extensive biodiversity screening
Source: Nat Commun. 2024 Jun 10;15:4933. doi: 10.1038/s41467-024-49009-2 (PMC11164908; doi:10.1038/s41467-024-49009-2)

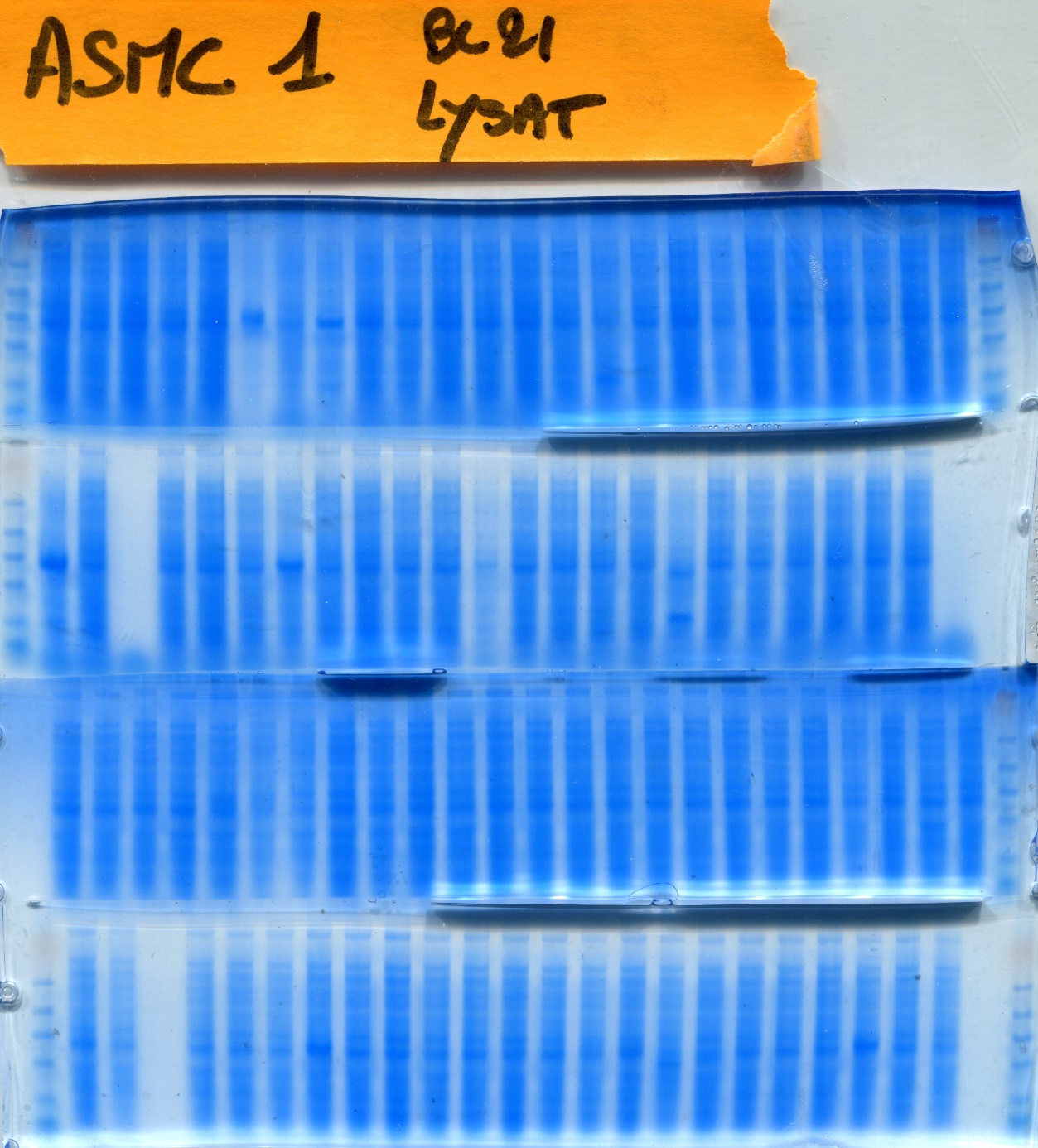


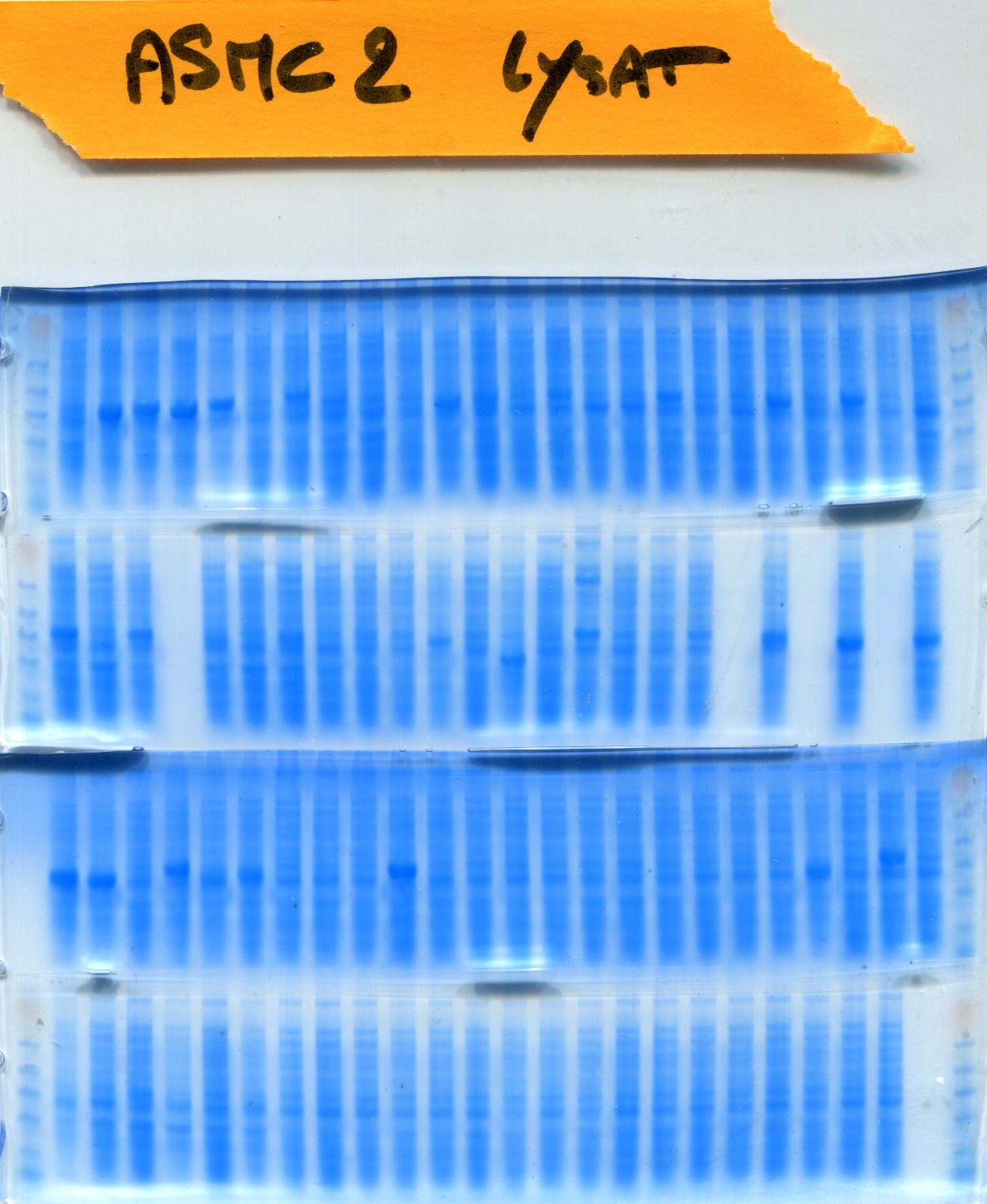


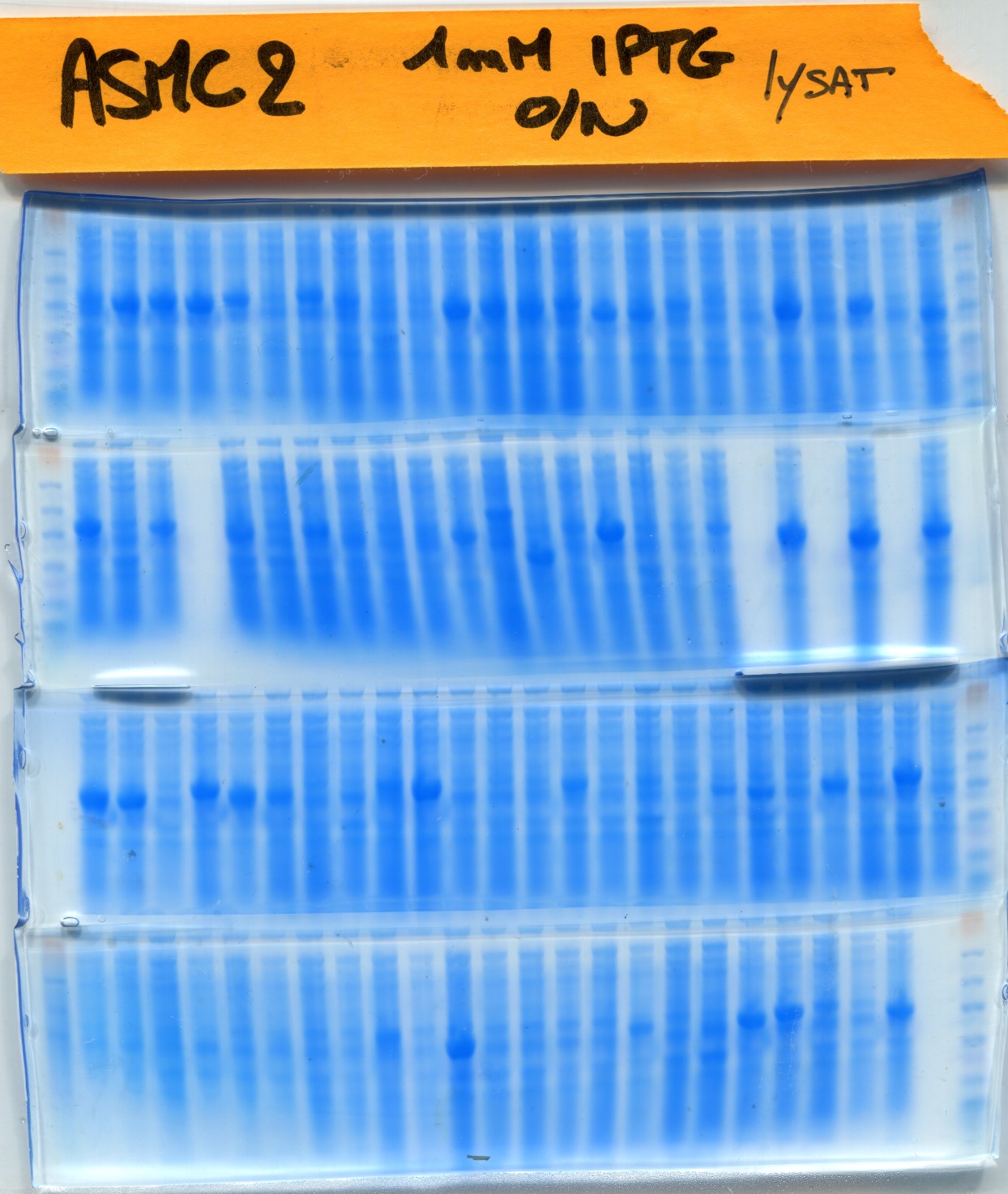


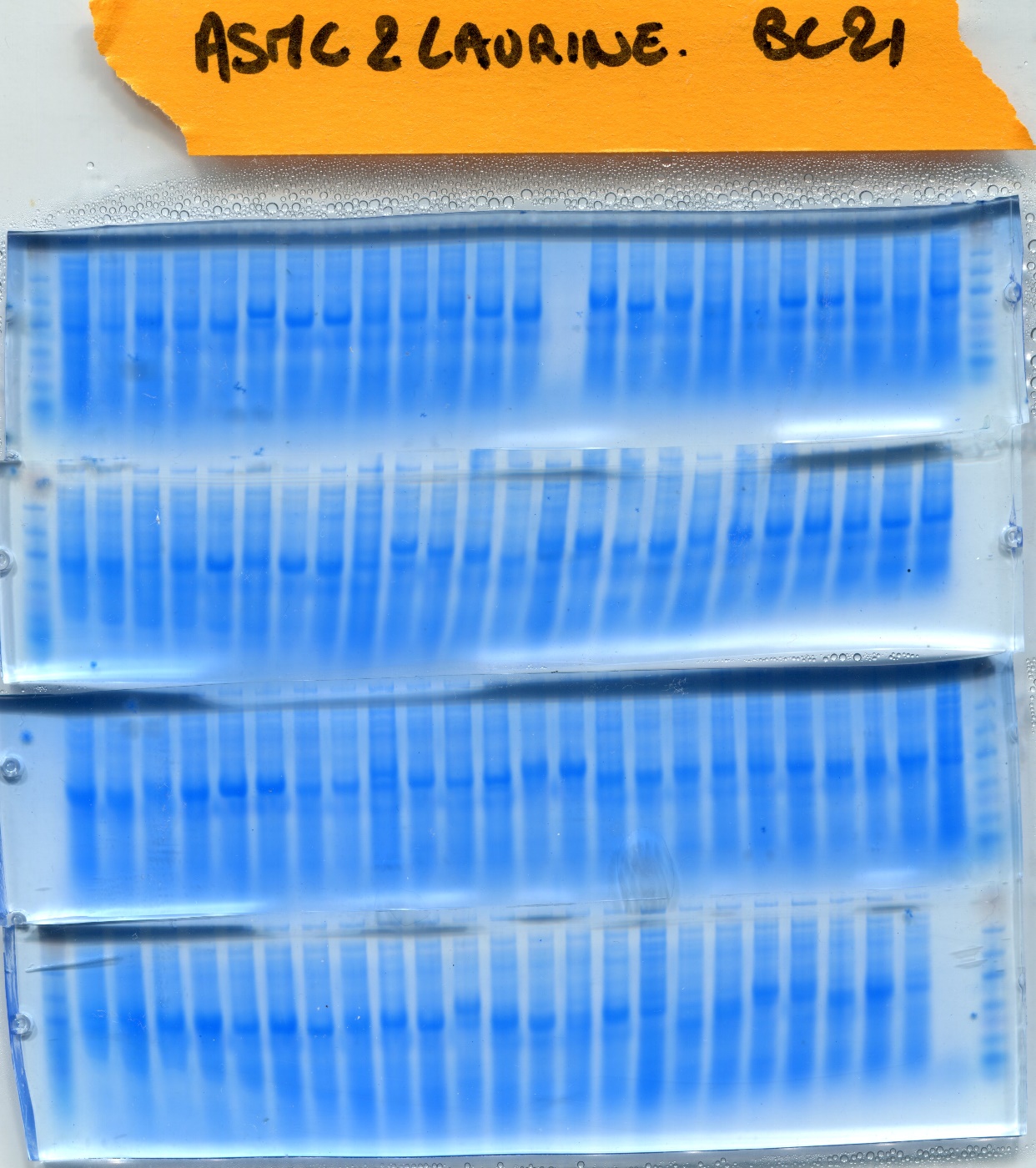


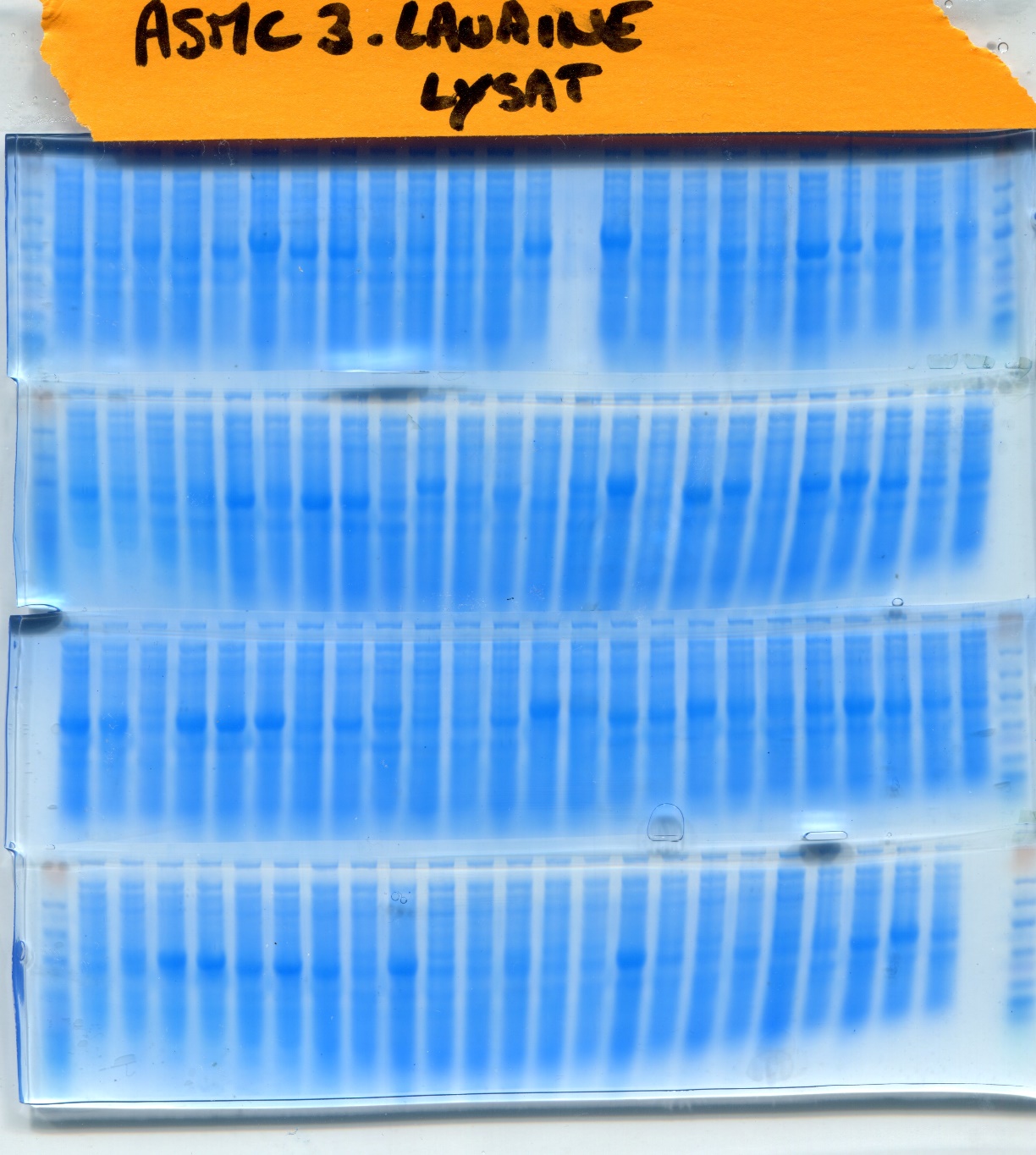


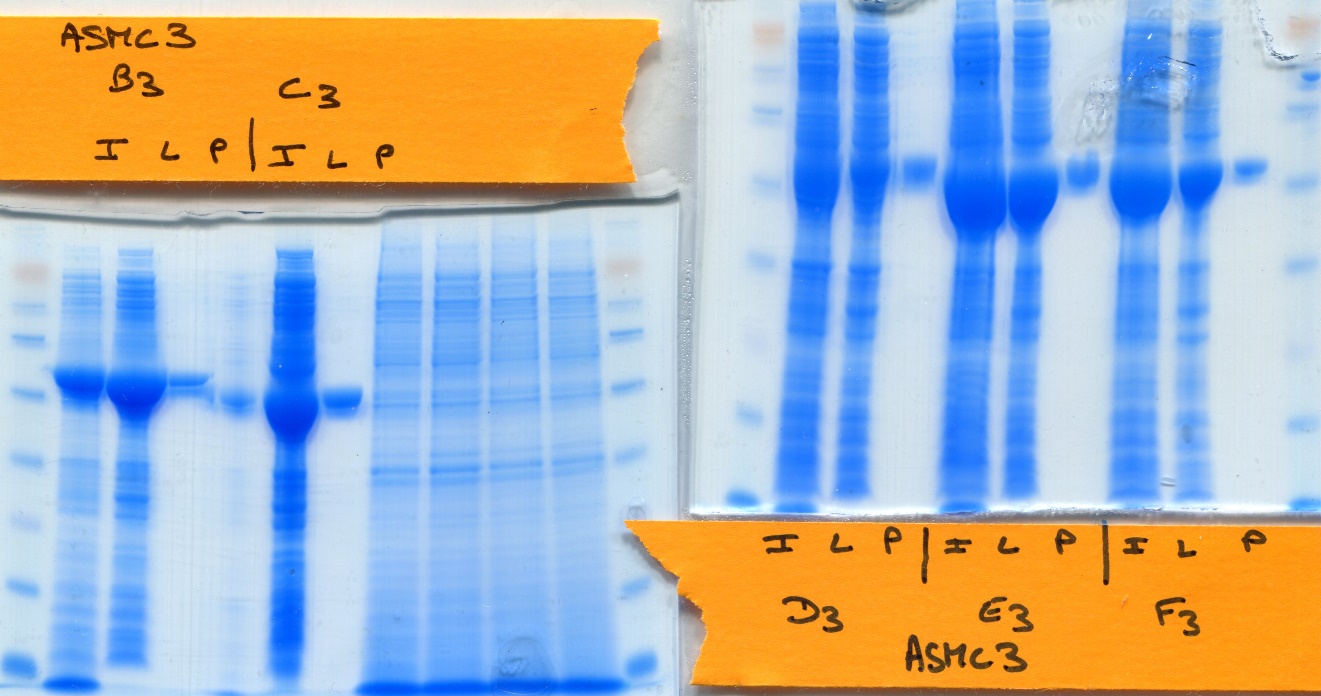


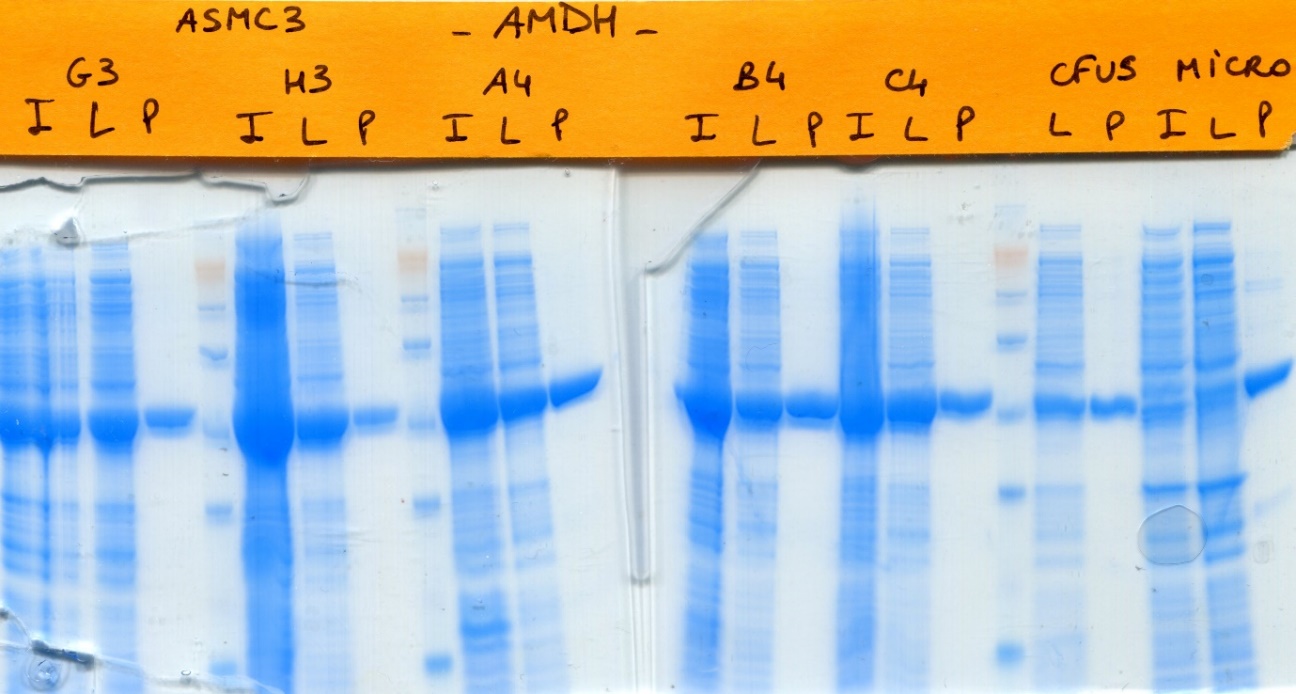


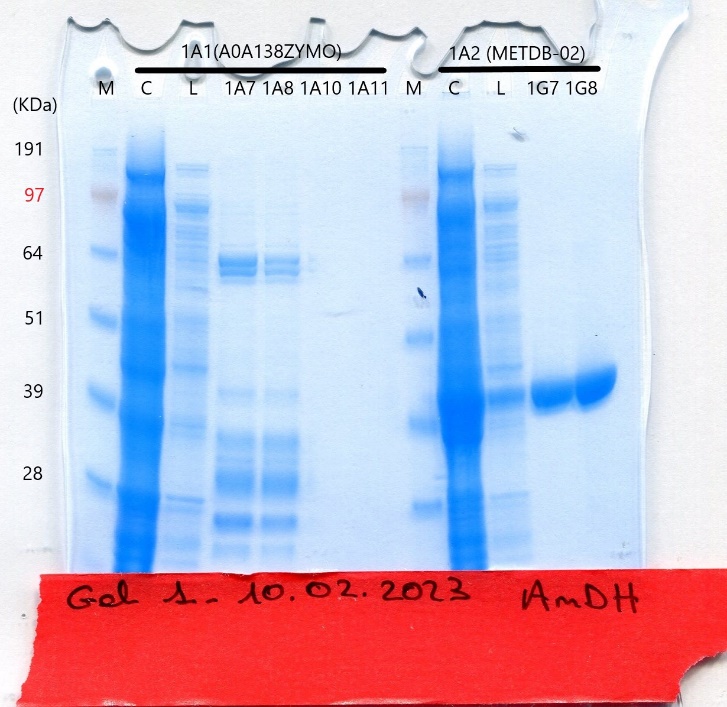


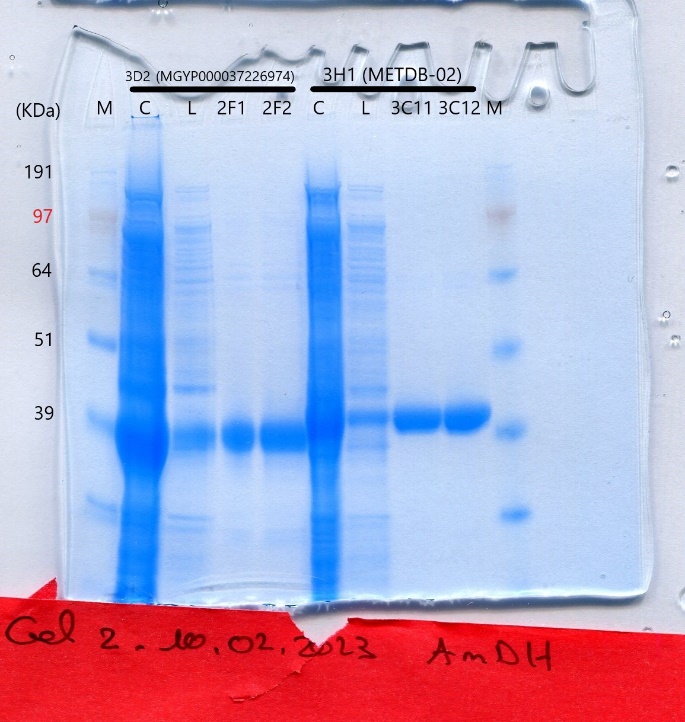


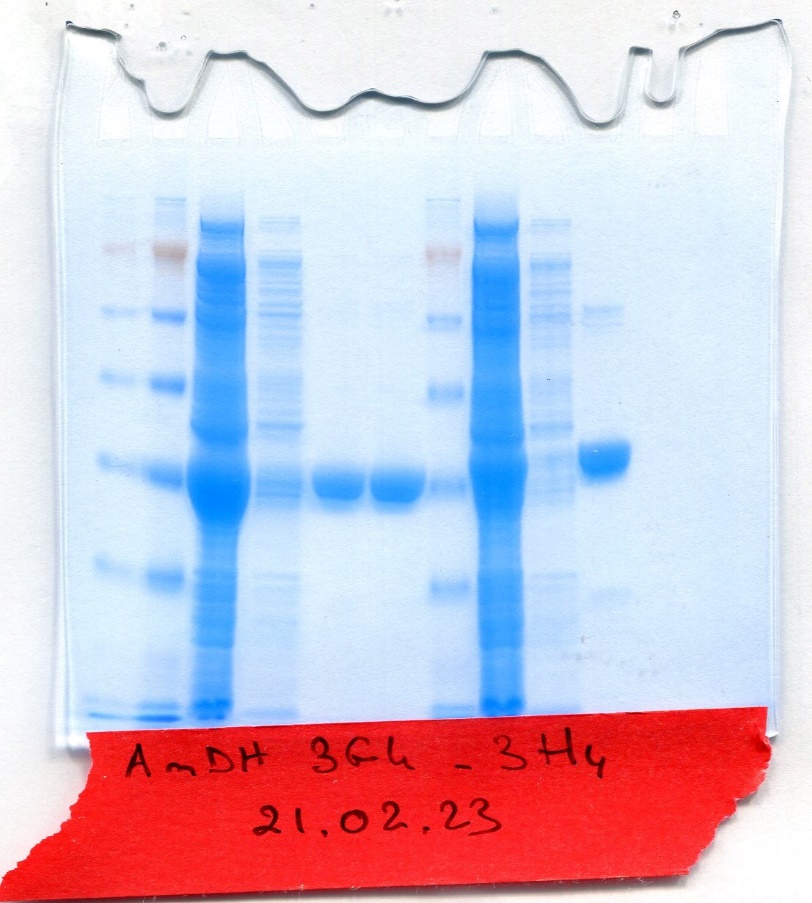


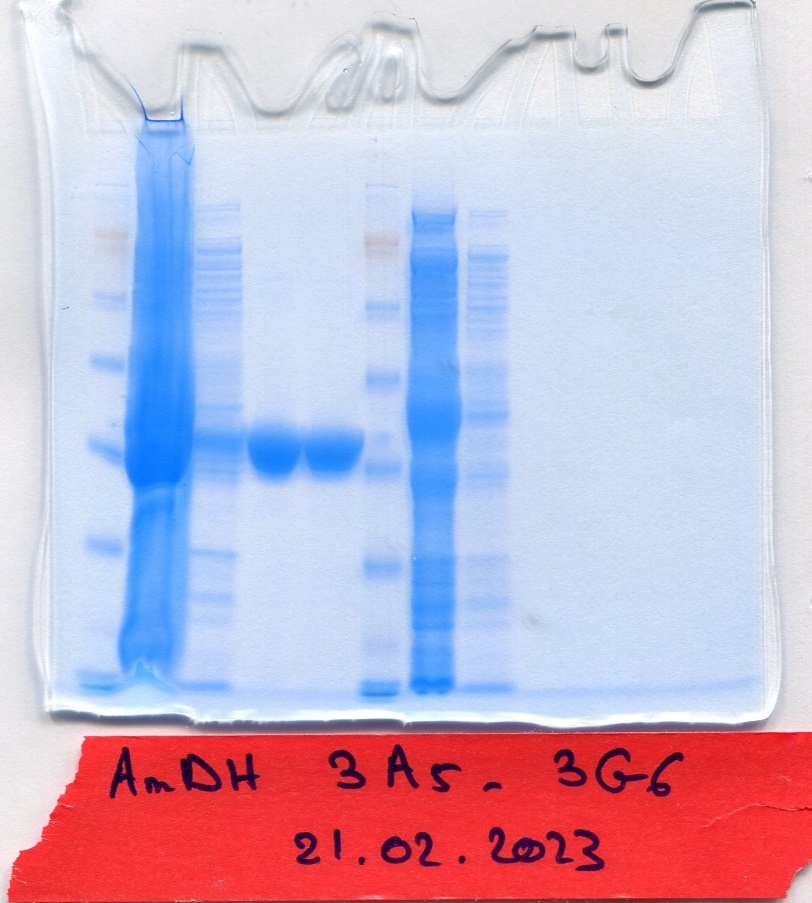


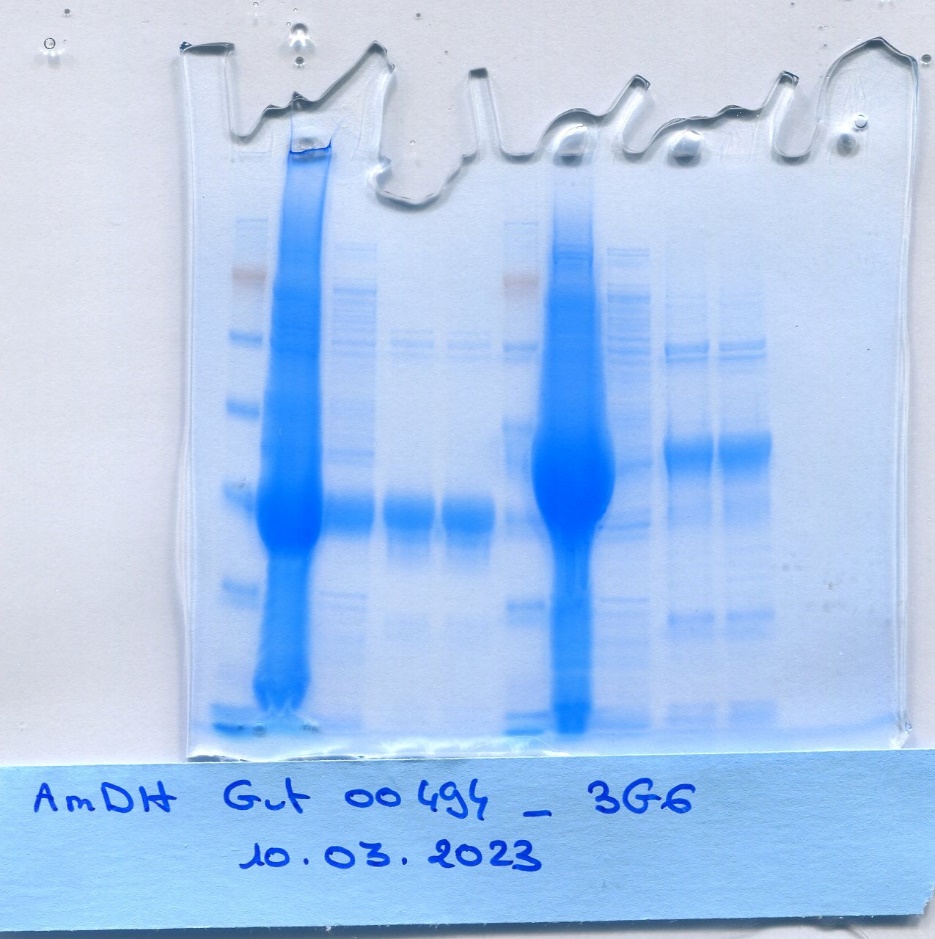


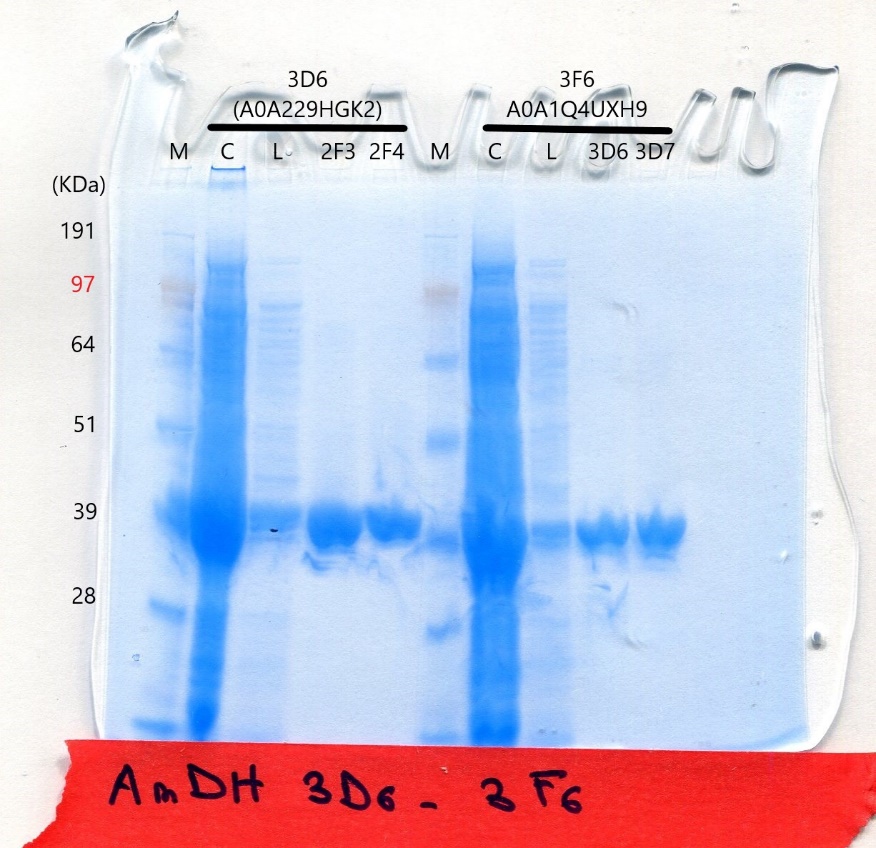


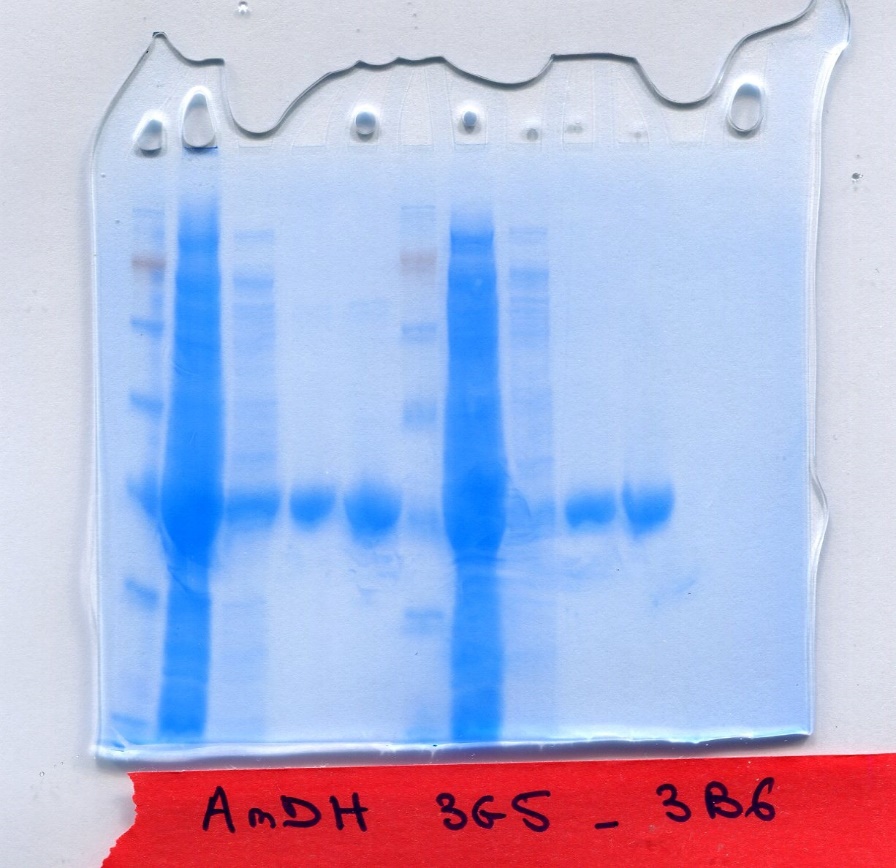


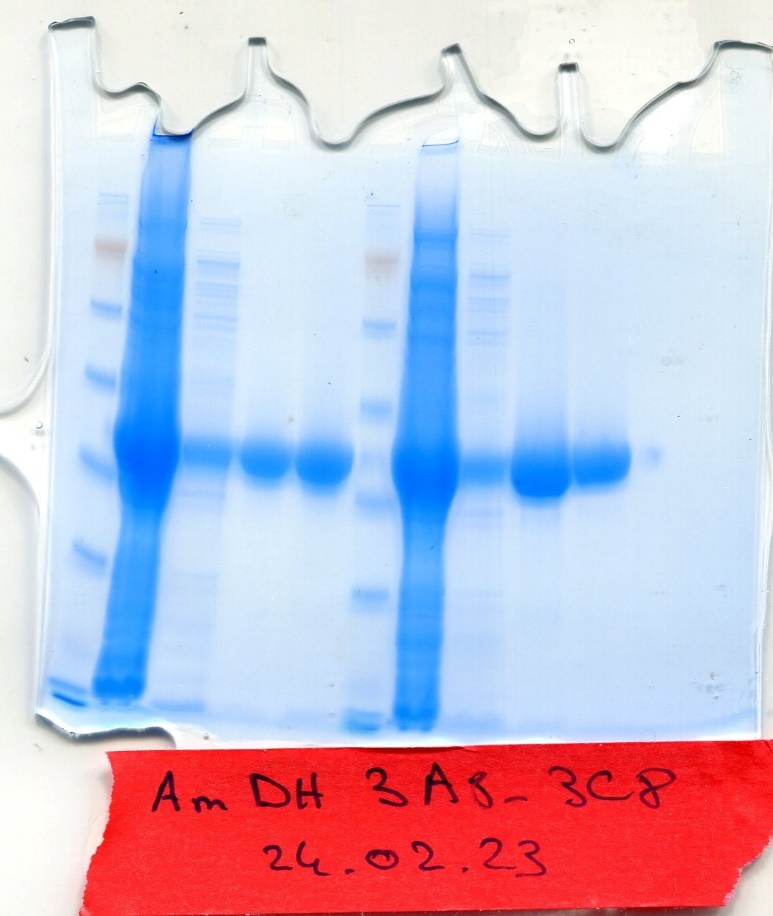


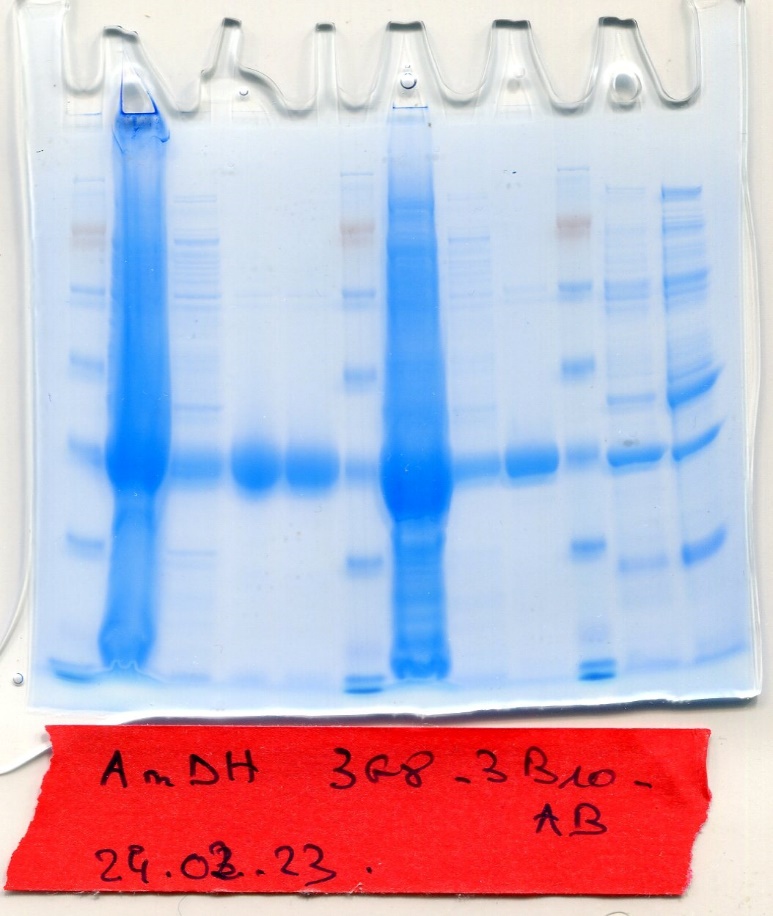


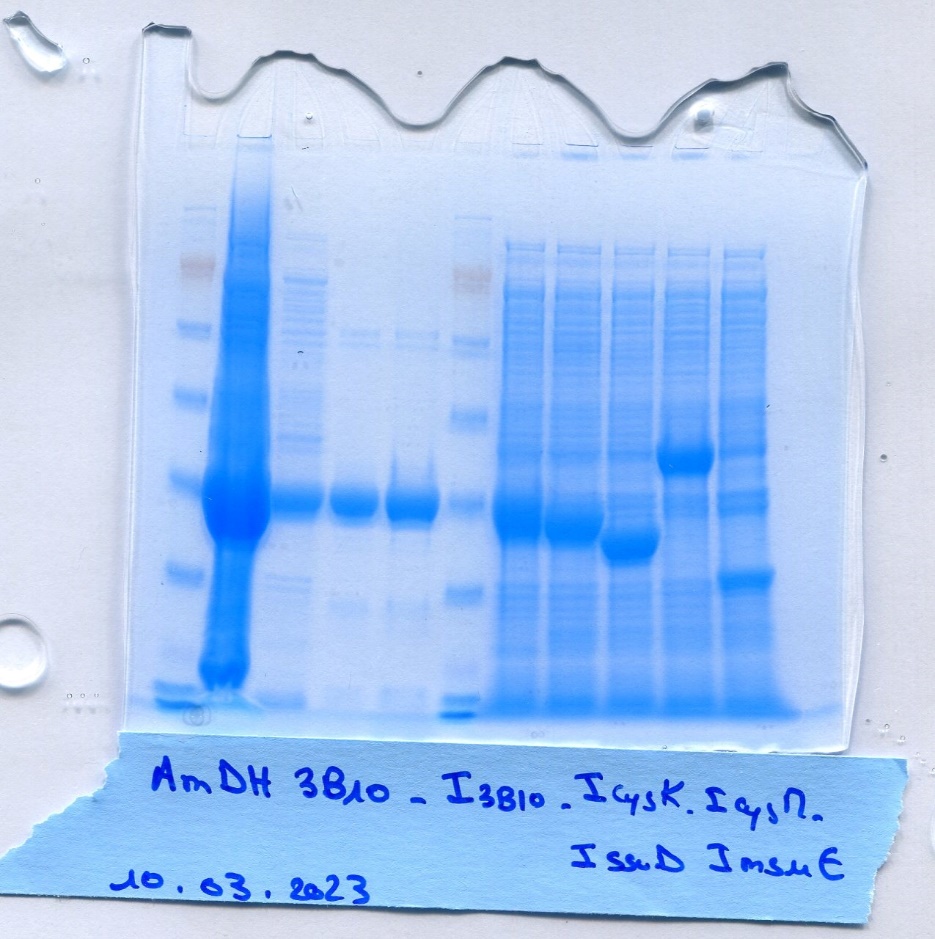

Supplement: Supplementary file 15 — Source Data [file 41467_2024_49009_MOESM15_ESM.zip › ZIP/source data Gel.docx]
